# Shared Causal Paths underlying Alzheimer’s dementia and Type 2 Diabetes

**DOI:** 10.1038/s41598-020-60682-3

**Published:** 2020-03-05

**Authors:** Zixin Hu, Rong Jiao, Panpan Wang, Yun Zhu, Jinying Zhao, Phil De Jager, David A. Bennett, Li Jin, Momiao Xiong

**Affiliations:** 1 State Key Laboratory of Genetic Engineering and Innovation Center of Genetics and Development, School of Life Sciences, Fudan University, Shanghai, China; 2 Department of Biostatistics and Data Science, School of Public Health, University of Texas Health Science Center at Houston, Houston, Texas USA; 3 Human Phenome Institute, Fudan University, Shanghai, China; 4 Department of Epidemiology, University of Florida, Florida, USA; 5 Center for Translational & Computational Neuroimmunology, Department of Neurology, Columbia University Medical Center, New York, 10033 USA; 6 Rush Alzheimer’s Disease Center, Rush University Medical Center, Chicago, IL 60612 USA

**Keywords:** Computational biology and bioinformatics, Genetics

## Abstract

Although Alzheimer’s disease (AD) is a central nervous system disease and type 2 diabetes MELLITUS (T2DM) is a metabolic disorder, an increasing number of genetic epidemiological studies show clear link between AD and T2DM. The current approach to uncovering the shared pathways between AD and T2DM involves association analysis; however such analyses lack power to discover the mechanisms of the diseases. As an alternative, we developed novel causal inference methods for genetic studies of AD and T2DM and pipelines for systematic multi-omic casual analysis to infer multilevel omics causal networks for the discovery of common paths from genetic variants to AD and T2DM. The proposed pipelines were applied to 448 individuals from the ROSMAP Project. We identified 13 shared causal genes, 16 shared causal pathways between AD and T2DM, and 754 gene expression and 101 gene methylation nodes that were connected to both AD and T2DM in multi-omics causal networks.

## Introduction

Although Alzheimer’s dementia is a central nervous system disease and type 2 diabetes MELLITUS (T2DM) is a metabolic disorder, an increasing number of epidemiological and genetic epidemiological studies show clear link between Alzheimer’s dementia and T2DM. Alzheimer’s dementia with great economic, political and social consequences is a progressive, irreversible degenerative disease of the brain and is the most common cause of dementia due to the gradual accumulation of amyloid-beta $(A\beta )$ and twisting of tau protein^1,2^, and other common brain pathologies^3^. Alzheimer’s dementia is also involved in inflammation and oxidative address and exhibits memory loss and cognitive dysfunction^4,5^.

Two mechanisms underlying T2DM are insulin resistance and insufficient insulin secretion from pancreatic *β*-cells^4^. T2DM patients are unable to process insulin signaling correctly. In response to insulin resistance, pancreatic *β*-cells increase insulin production. However, when pancreatic *β*-cells gradually lose function; insulin production cannot be increased to maintain normal glucose levels. The brain is a target organ for insulin^6^. Insulin signaling plays an important role in the organization and function of the brain and impaired insulin signaling induces an overactivation of GSK-3 kinase, increases tau phosphorylation, alters tau modification and neurofibrillary degeneration^7^. T2DM also suffer from mild to severe nervous system damage. Persistent blood glucose may impair blood flow to the brain^8^.

Prior work in ROSMP found an association of T2DM with incident Alzheimer’s dementia and rate of cognitive decline^9^. However, we did not find an association with Alzheimer’s disease (AD) pathology^10^. Rather, we found an association with cerebral infarcts. Other evidence from ROSMP continue to point to potential common mechanisms. For example, we found that brain insulin signaling was associated with AD pathology^11^. We also found interactions between *GSKβ* polymorphisms associated with β-amyloid deposition^12^.

The current approaches to identifying several shared pathophysiology processes between Alzheimer’s dementia and T2DM have several limitations. Firstly, the most previous works have focused on identifying biological pathways underlying AD and T2DM. Few attempts to discover the role of dysregulated SNPs, gene expressions and methylations have been carried out. Secondly, the conventional evidences for linking AD and T2DM purely depend on the statistical association^13^. There has been increasing recognition that association and causation are different concepts^14^. Association attempts to measure dependence between two variables, while causation is to study the distribution of the variable (effect) after taking action on the another variable (cause). The statistical tool for association analysis is the conditional distribution, while the tool for the causal analysis is the intervention calculus. Many association signals may not be causal signals and some causal signals may not show strong association. If causation loci were searched only from association loci, many causation loci might be missed. The widely used gene expression networks are co-expression networks and phenotype networks are correlation networks. The major tools for integrated omics analysis are based on association analysis. The networks in the most multilevel omics analysis are undirected graphs. It is difficult to use undirected graphs for identifying the causal paths from genetic variants to diseases.

We are facing a great challenge to shift the current analytic platforms of genetic analysis from genetic association analysis to multilevel omics causal analysis for unraveling the mechanic link between AD and T2DM. To meet this challenge, we need (1) to develop and implement causation analysis methods for genetic studies of AD and T2DM; (2) to develop a general framework for construction of multilevel causal omics networks to discover common paths from genetic variations to AD and T2DM via methylations, gene expressions and multiple phenotypes. The real dada set ROSMAP^15,16^ will be used to valid the multilevel omics causal networks as a useful analytic platform for identifying shared causal paths between AD and T2DM and demonstrates that the proposed methods are capable of identifying the shared pathologic paths between AD and T2DM. A program for construction of multilevel causal networks can be downloaded from https://github.com/wenrurumon/mysrc/tree/master/CNIF_0.3.0.

## Results

### Simulations

To evaluate the performance of the proposed causal network analysis, we conducted a series of simulation studies to compare the detection power and false discovery rate (FDR) for three methods: (1) weighted gene co-expression network (WGCNA), (2) structural equation model (SEM) and structural equation model coupled with integer programming (SEMIP).

We randomly generated 1,000 directed acyclic graphs (networks) with 20 nodes (15 gene expression or phenotype nodes and 5 genotype nodes) and mean 30 directed edges, 1,000 directed acyclic graphs (networks) with 30 nodes (22 expression/phenotype nodes, 8 genotype nodes), and mean 47 directed edges, and 40 nodes (30 gene expression or phenotype nodes and 10 genotype nodes), and mean 68 directed edges, respectively. Simulation results were summarized in Table 1 where we only listed undirected network results because the WGCNA can only estimate the undirected network. We calculated the power and FDR of three methods for 100, 300, 500 and 1,000 samples. We can observe that in all cases, The SEMIP had the largest power and smallest FDR. When the number of nodes in the networks increased, the power to identify the structure of the networks decreased, while FDR increased. When the number of nodes reached 40, the SEMIP can reach 68.5% power and 7.40% FDR using 1,000 samples.

**Table 1:** Power and FDR of three methods for construction of causal networks with 20,30 and 40 nodes.

Methods	Nodes	Sample Sizes	Undirected		Directed	
			Power	FDR	Power	FDR
WGCNA	20	100	51.60%	16.00%		
WGCNA	20	300	53.00%	15.30%		
WGCNA	20	500	66.40%	13.00%		
WGCNA	20	1000	82.60%	13.60%		
SEM	20	100	70.80%	44.40%	50.70%	42.00%
SEM	20	300	77.80%	49.20%	53.90%	23.20%
SEM	20	500	83.50%	46.40%	56.90%	44.90%
SEM	20	1000	98.20%	32.40%	57.30%	26.70%
SEMIP	20	100	64.60%	34.70%	59.50%	15.50%
SEMIP	20	300	73.50%	39.30%	65.40%	17.40%
SEMIP	20	500	77.60%	25.40%	68.30%	12.00%
SEMIP	20	1000	86.60%	22.60%	76.60%	13.20%
WGCNA	30	100	43.30%	21.10%		
WGCNA	30	300	49.80%	15.00%		
WGCNA	30	500	53.60%	21.00%		
WGCNA	30	1000	56.50%	13.20%		
SEM	30	100	64.30%	34.10%	46.70%	26.00%
SEM	30	300	73.60%	41.20%	49.30%	22.50%
SEM	30	500	82.30%	34.80%	52.40%	34.10%
SEM	30	1000	94.50%	36.30%	52.80%	27.60%
SEMIP	30	100	63.30%	15.50%	58.50%	16.40%
SEMIP	30	300	67.40%	27.00%	63.50%	13.50%
SEMIP	30	500	71.50%	18.30%	64.20%	10.80%
SEMIP	30	1000	94.80%	28.60%	71.80%	15.00%
WGCNA	40	100	43.30%	21.40%		
WGCNA	40	300	49.20%	17.00%		
WGCNA	40	500	51.40%	19.70%		
WGCNA	40	1000	54.10%	18.20%		
SEM	40	100	61.70%	37.30%	46.50%	29.50%
SEM	40	300	70.10%	25.70%	49.60%	38.20%
SEM	40	500	79.90%	35.30%	54.50%	17.70%
SEM	40	1000	95.10%	45.90%	62.60%	27.90%
SEMIP	40	100	62.70%	23.20%	58.30%	11.60%
SEMIP	40	300	64.50%	21.10%	62.10%	10.30%
SEMIP	40	500	75.50%	32.30%	66.20%	15.30%
SEMIP	40	1000	82.00%	34.00%	68.50%	7.40%

### Shared genetic loci underlying AD and T2DM

The number of AD and T2DM directly connected or indirectly connected genes was summarized in Table 2. The total number of genes connected to both AD and T2DM including directly connected and indirectly connected was 759. The genes that were both directly and indirectly connected to both AD and T2DM were summarized in Table S1. The genes that were indirectly connected to AD and both directly and indirectly connected to T2DM were listed in Table S2. Similarly, the genes that were both directly and indirectly connected to AD and indirectly connected to T2DM were summarized in Table S3.

**Table 2:** The number of genes connected to AD and T2DM.

		To T2DM			
		Directly Connected	Indirectly Connected	Both Directly and Indirectly Connected	Not Connected
To AD	Directly Connected	5			13
	Indirectly Connected		682	13	
	Both Directly and Indirectly Connected		20	8	
	Not Connected	17			

We also tested causation of 299 pathways in the KEGG pathway database to AD and T2DM (Described in detail in the Methods section). The results were summarized as follows. The number of pathways that were directly connected to both AD and T2DM was 16; the number of pathways that were directly connected to AD and indirectly connected to T2DM was 17; the number of pathways that were directly connected to T2DM and indirectly connected to AD was 18, the number of pathways that were indirectly connected to both AD and T2DM was 114; the number of pathways that were directly connected to AD and not connected to T2DM was 6; the number of pathways that were not connected to AD and directly connected to T2DM was 2.

Then, we investigated shared gene expressions via multilevel causal networks. We summarized the results as follows. The number of expression genes that were directly connected to both AD and T2DM was two genes: GRMD1B, RP1-111D6.3, the number of expression genes that were directly connected to AD, but not directly connected to T2DM was 19 (P-value < 10^−4^, Table S4**)** and the number of expression genes that were directly connected to T2DM, but not directly connected to AD was 7 (P-value < 10^−4^, Table S5). The number of expression genes that were indirectly connected to both AD and T2DM was 725.

Similarly, we can study shared methylation via multilevel causal networks. The number of methylated sites/ genes that were directly connected to AD, but not directly connected to T2DM was 17 (Table S6) and the number of methylated sites/genes that were directly connected to T2DM, but not directly connected to AD was 27 (Table S7). The number of methylated sites/genes that were indirectly connected to both AD and T2DM was 117 (Table S8).

The number of phenotypes that were directly connected to both AD and T2DM was six (Age, CHL, HDL ratio, LDL, Semantic memory and working memory).

### Shared CREBBP, MAPK and PI3K-AKT pathways between AD and T2DM

**Figure 1:**
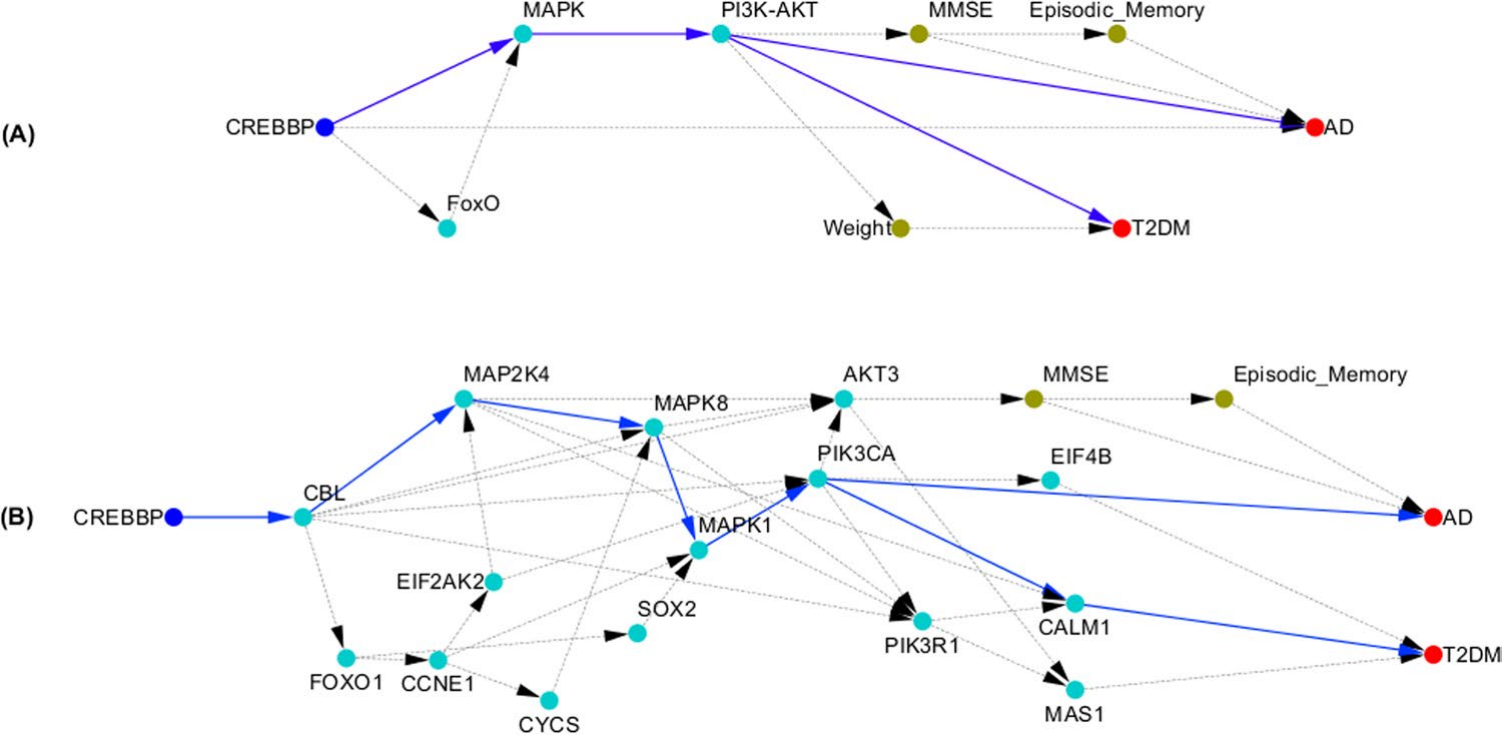
(**A**) Shared CREBBP, MAPK and PI3K-AKT pathways between AD and T2DM; (**B**) Shared causal subnetwork structure from CREBBP to AD and T2DM.

To assess whether CREBBP is a common genetic factor of AD and T2DM, and how CREBBP mediates the development of AD and T2DM, we searched the all possible paths from gene CREBBP to AD and T2DM in the inferred multilevel causal network. The results were shown in Fig. 1. Figure 1A plotted the path from CREBBP to AD and T2DM via MAPK and PI3K-AKT signaling pathways. The genes in the MAPK and PI3K-AKT signaling pathways, CREBBP, episodic memory, MMSE, AD and T2DM were then used to further infer causal networks using SEMs and IP. The inferred causal network was shown in Fig. 1B. From Fig. 1B we observed a path from CREBBP to AD and T2DM via gene connections: $CREBBP\to CBL\to MAP2K4\to MAPK8\to MAPK1\to PIK3CA$. MAPK and PI3K-AKT pathways play critical roles in memory.

### Shared TTC3, FoxO, MAPK, and PI3K-AKT Pathways between AD and T2DM

Next we presented an example to illustrate shared causal paths that started a gene directly connected to AD and indirectly connected to T2DM.

**Figure 2:**
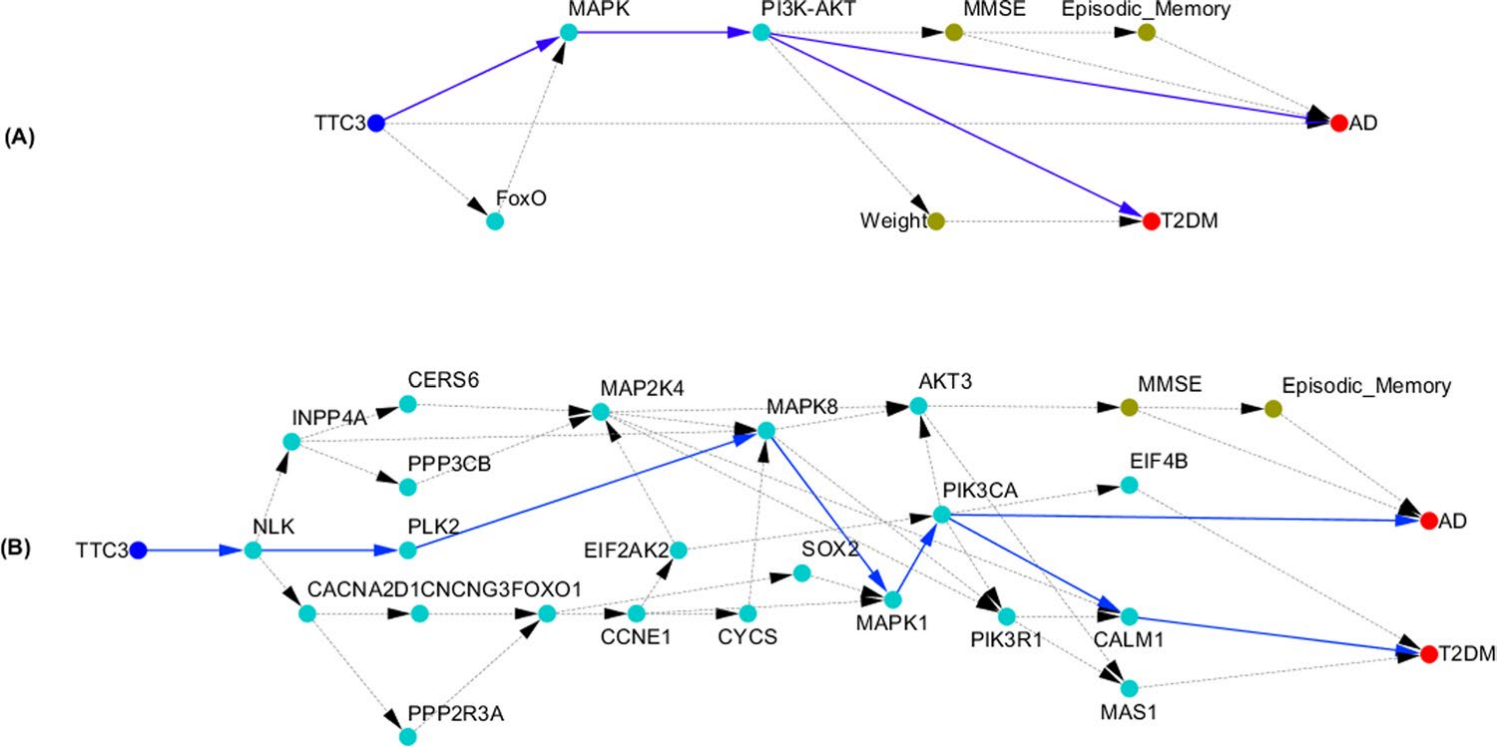
(**A**) Shared TTC3, FoxO, MAPK, and PI3K-AKT Pathways between AD and T2DM; (**B**) Shared causal subnetwork structure from TTC3 to AD and T2DM.

Again, we used the DFS algorithm to search the causal paths from multilevel causal networks. The causal paths from TTC3 to AD and T2DM were shown in Fig. 2. The paths from MAPK and PI3K-AKT pathway to AD and T2DM were the same as that in Fig. 1. The genes in the FoxO, MAPK and PI3K-AKT signaling pathways, TTC3, and episodic memory, MMSE, weight, AD and T2DM were then used to further infer causal networks using SEMs and IP. The structure of the inferred network was shown in Fig. 2B. There were a large number of causal paths from *TTC3* to either AD or T2DM. The shared common causal paths were $$TTC3\to NLK\to CACNA2D1\to CNCNG3\to FOXO1$$$$\to CCNE1\to CYCS\to MAPK1\to PIK3CA$$ and $$TTC3\to NLK\to PLK2\to MAPK8\to MAPK1\to MAPK1\to PIK3CA$$.

### Shared morphine addiction and neuroactive ligand receptor interaction pathways

Searching the causal paths from gene *HNF4G* to AD and T2DM via the multilevel causal networks using the DFS algorithm, we found that *HNF4G* was indirectly connected to AD and T2DM. In addition to shared MAPK and PI3K-AKT pathways between AD and T2DM which were discussed in the previous sections, we observed shared two new pathways between AD and T2DM: morphine addiction and neuroactive ligand receptor interaction pathways as shown in Fig. S1A. The structure of the inferred network that consisted of shared morphine addiction and neuroactive ligand receptor interaction pathways between AD and T2DM was shown in Fig. S1B. There were more than 10 shared causal paths. We observed two shared major causal paths: (1) $HNF4G\to NLK\to PLK2\to MAPK8\to MAPK1\to PIK3CA\to AKT1$ amd (2) $HNF4G\to NLK\to GNGT2\to PLCB2\to PLCB1\to ADRB1$.

### Shared fatty acid biosynthesis and primary bile acid biosynthesis pathways

**Figure 3:**
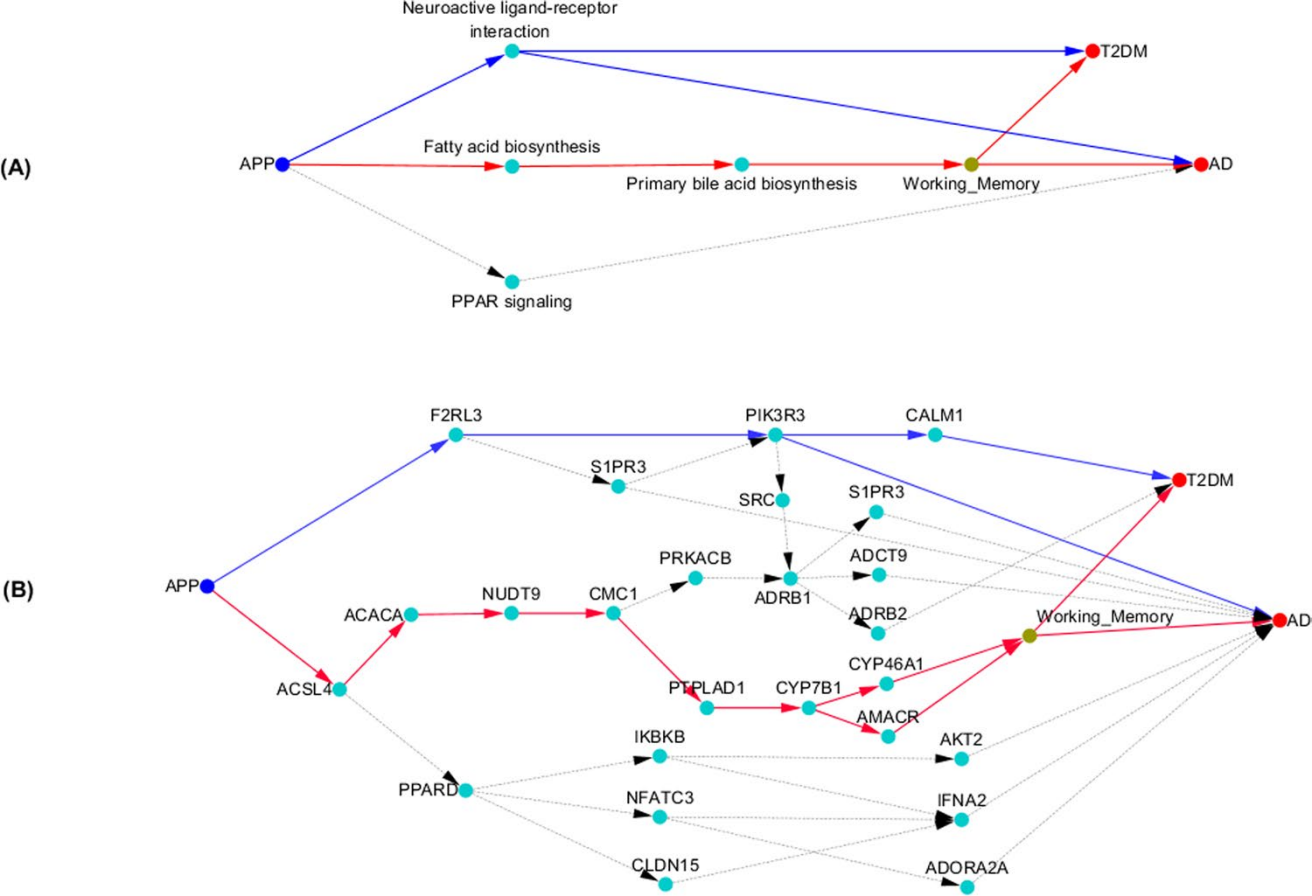
(**A**) Shared APP, Fatty Acid Biosynthesis and Primary Bile Acid Biosynthesis Pathways between AD and T2DM; (**B**) Shared causal subnetwork structure from APP to AD and T2DM.

Our data also provided evidence to show that fatty acid biosynthesis and primary bile acid biosynthesis pathways were shared pathways between AD and T2DM. Searching the multilevel causal networks from APP to AD and T2DM using the DFS algorithm, we identified the shared causal paths from APP to both AD and T2DM, shown in Fig. 3A. There were two shared causal paths between AD and T2DM: $APP\to \,neuroactive\,ligand\,receptor\,interaction\,$ and $APP\to fatty\,acid\,biosynthesis\to primary\,bile\,acid\,biosynthesis$. Neuroactive ligand receptor interaction pathway was discussed in the previous section.

Next we presented the causal network structure of the shared genes between AD and T2DM in the two shared causal paths in Fig. 3B. We observed two major shared paths from APP to AD and T2DM. One path was *APP* → *ACSLA* → *ACACA* → *NUDT9* → *CMC*1 → *PTPLAD*1 → *CYP*781 → *CYP*46*A*1 → *working memory* (*or CYP*781 → *AMACA* → *working memory*). Another causal path was *APP* → *F2RL3* → *PIK3R3* → (*or F2RL3* → *S1PR3* → *PIK3R3*.

To further illustrate the validity of the inferred causal paths, we presented Fig. S2 that showed the average levels of expression of the genes in Fig. 3 for AD, T2DM and normal individuals. From Fig. 3, Figs. S2 and S3, we can observed that the genes along the path $APP\to F2RL3\to PIK3R3\,(or\,F2RL3\to S1PR3\to PIK3R3)$ of the individuals with AD were over expressed, and the genes along the path *APP* → *ACSLA* → *ACACA* → *NUDT9* → *CMC*1 → *PTPLAD*1 → *CYP*781 → *CYP*46*A*1 → *working memory* (*or CYP*781 → *AMACA* → *working memory*) of the individuals with AD were under expressed. Genetic variation in gene *APP* either regulated over expressed genes or regulated under expressed genes. Both of them caused AD. For the individuals with T2DM, the majority of gene expressions along the causal paths from *APP* to T2DM which were regulated by genetic variation in gene *APP* was under expressed.

### Shared methylated genes POU3F2, KIF4B and TNSL3, and dopaminergic synapse and AMPK pathways

**Figure 4:**
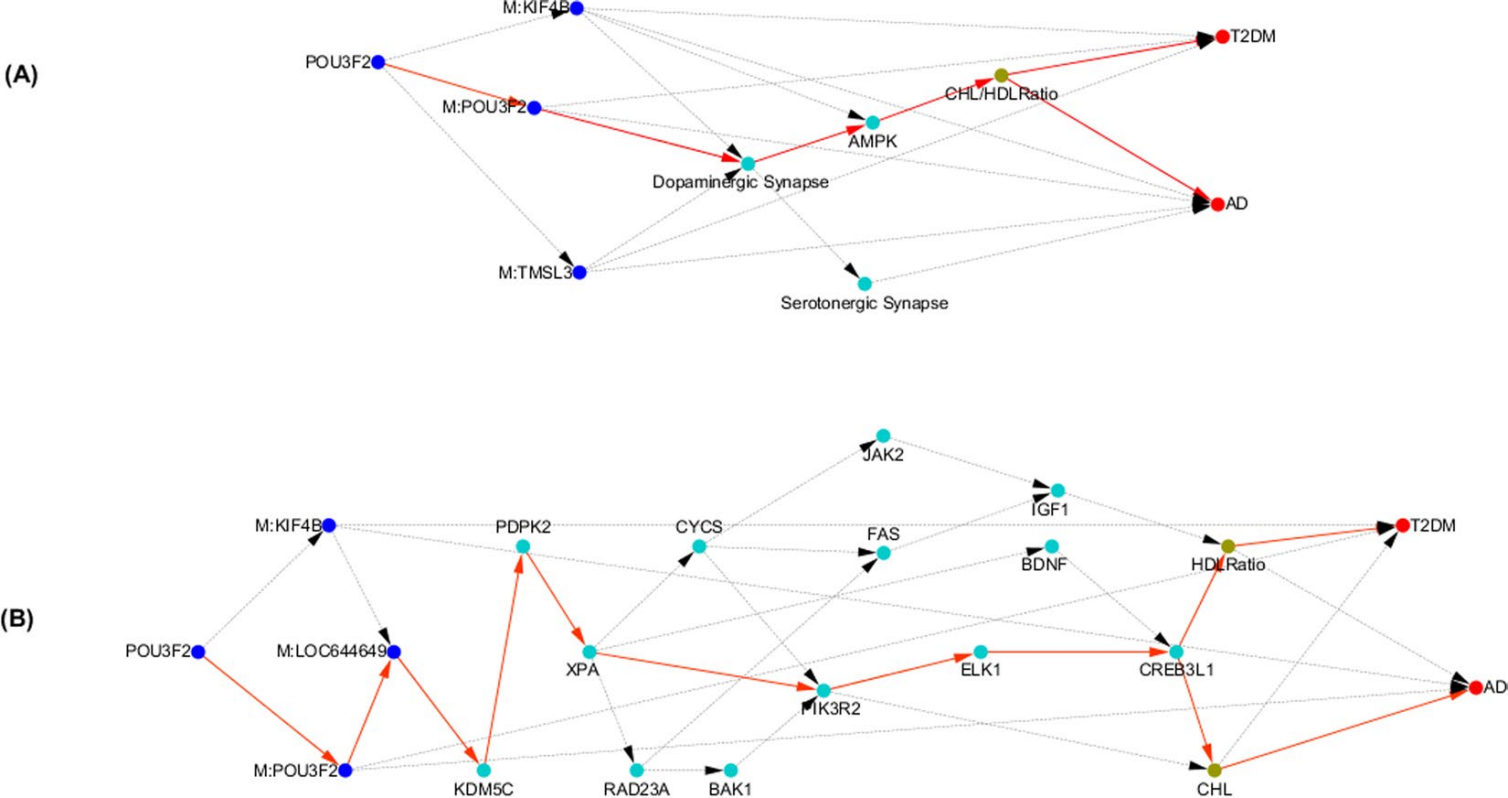
(**A**) Shared Methylated Genes POU3F2, KIF4B and TNSL3, and Dopaminergic Synapse and AMPK Pathways between AD and T2DM; (**B**) Shared causal subnetwork structure from POU3F2 to AD and T2DM.

In this section, we illustrate how a shared gene regulates three shared gene methylations, which in turn regulate the shared pathways. Our results showed that genetic variation in gene *POU3F2* regulated gene expressions in dopaminergic synapse and AMPK pathways via methylations of *POU3F2*, *KIF4B* and *TMSL3*, which in turn influences CHL/HDL Ration, and finally led to AD and T2DM (Fig. 4A).

Again, we presented the causal network structure of the shared genes between AD and T2DM in the two shared dopaminergic synapse and AMPK pathways in Fig. 4B. There were multiple shared directed paths from POU3F2 to AD and T2DM. A major shared directed path: *m*: *POU*3*F*2 → *m*: *LOC*644649 → *KDM*5*C* → *PDPK*2 → *XPA* → *MK*3*R*2 → *ELK*1 → *AD* (*or* → *CHL* → *T*2*DM*).

## Discussion

This papers addresses several issues for uncovering causal paths shared between AD and T2DM. The first issue is to shift the current paradigm of genetic analysis from association analysis to deep causal inference for uncovering the shared mechanisms between AD and T2DM. The current paradigm for discovering mechanisms of diseases is association analysis. There is increasing recognition that a large proportion of association signals are not causal signals and causal signals may not be association signals. A large number of causal signals cannot be derived from set of association signals. Only searching causal signals from association analysis, a large proportion of causal signals will be missing. Therefore, the ANMs were developed as practical causal inference methods to identify the genetic variants that cause disease.

Second issue is to shift the current paradigm of genetic analysis from genetic analysis alone to integrated causal genomic, epigenomic, transcriptional and phenotypic data analysis for unraveling the mechanisms of AD and T2DM. The widespread existing omics networks that are essentially undirected graphs. Using undirected graphs, we cannot to identify direct causal relations among diversified types of variables at multilevel and the causal routes from genetic variants to complex phenotypes via omics. In this paper, we develop novel statistical methods for multilevel causal omics network construction and provide pipelines for uncovering shared causal paths between AD and T2DM via gene expressions, DNA methylations, environments and multiple phenotypes.

The third issue is to develop algorithms that can automatically search the causal routes from genetic variations to the complex phenotypes. The size of multilevel causal omics network is large. The number of nodes of such networks can reach ten thousand. The number of causal paths is huge. Manually searching causal paths from large causal networks is infeasible. To meet the challenge of searching causal paths from large causal networks, we develop computer representation of large causal networks and algorithms for searching the causal paths.

The results of application of the proposed pipelines for identifying causal paths to real data analysis of AD and T2DM provided strong evidence to support the link between AD and T2DM and unraveled causal mechanism to explain this link. We identified the shared causal genes, gene expressions, DNA methylations and pathways between AD and T2DM. Some of them can be supported by literature and some of them are new.

Specifically, we identified the shared CREBBP, MAPK and PI3K-AKT pathways between AD and T2DM. Binding of transcription factors to the cyclic Adenosine Monophosphate (cAMP) response element (CRE) regulates the activity of RNA polymerase. cAMP Response Element binding protein (CREB) is a cellular transcription factor that binds the CRE^17^. CREB-binding protein (CREBBP) and CREB together mediate the conversion of short-term memory to long-term memory and alternate the activity of the β-amyloid (Aβ) peptide, which in turn regulates hippocampal-dependent synaptic plasticity^18,19^. Cognitive function such as working memory is involved in insulin signaling dysfunction and blood glucose levels. It was reported that working memory is linked with T2DM^20–22^.

The shared TTC3, FoxO, MAPK, and PI3K-AKT Pathways between AD and T2DM were also identified. The tetratricopeptide repeat domain 3 (TTC3) gene was an AD causing gene (P-value for causation of AD < 0.0001), but not directly connected to T2DM (P-value for causation of T2DM = 0.47). TTC3 is associated with differentiation of neurons^23^. It is reported that a rare TTC3 variant is related with AD^24^. The TTC3–RhoA pathway could be a key determinant of the neuronal development, resulting in detrimental effects on the normal differentiation program^25^. Rho regulates the activation of MAPK pathway^26^. The Forkhead box O (FoxO) transcription factors that affect nervous system amyloid (Aβ) production, are implicated in the regulation of cell apoptosis and survival, and accelerate the progression of degenerative disease. FoxO pathway is involved in the PI3K/Akt and mitogen-activated protein kinase (MAPK) pathways in neuronal apoptosis in the brain.

FoxOs also can offer protection in the nervous system, reduce toxic intracellular protein accumulations and potentially effect Aβ toxicity^19,27,28^. Akt-FoxO that suppresses TLR4 signaling in Human Leukocytes is implicated in the development of T2DM^29^. Increasing evidences indicate that PI3K/AKT pathway are implicated in the development of T2DM^30,31^.

Our results further supported that morphine addiction and neuroactive ligand receptor interaction pathways were shared between AD and T2DM. Morphine addiction has neurotoxic effects and damages to the brain regions that function for learning, memory and emotions^32^. High dose of morphine may increase risk to T2DM^33^. It is also reported that neuroactive ligand receptor interaction pathway is associated with both AD and T2DM^34^.

The causal network analysis provided evidence that fatty acid biosynthesis and primary bile acid biosynthesis pathways were shared between AD and T2DM. Brain function such as intelligence, memory, behavior and concentration are all influenced by brain nutrition^35^. Omega-3 fatty acids affect the fluidity of brain cell membranes, neurotransmitter synthesis and signal transmission and are implicated in AD^36,37^. Bile acids are involved in cell signaling and immune function. It performs as potent inhibitors of apoptosis and regulates transcriptional and post-transcriptional events that affect mitochondrial function in neurons^38^. A trend of increased bile acids in AD has been observed^39^. Fatty acid utilization induces insulin resistance^40^. Bile acids are signal molecules and play an important role in regulating metabolism and inflammation. The abnormal bile acids are correlated with changes in insulin secretion, which lead to T2DM^41,42^. The amyloid precursor protein (APP) is a transmembrane protein. The aggregated amyloid-β (Aβ) peptides are generated by sequential proteolytic processing of the APP. Accumulation of Aβ and the APP play an important role in regulating lipid homeostasis including fatty acids, which finally affect the development of AD^43^.

Finally, we showed that how the causal analysis identified the shared methylated genes POU3F2, KIF4B and TNSL3, and dopaminergic synapse and AMPK pathways between AD and T2DM. Emerging evidences indicate that methylation alternations to DNA of the brain are linked to Alzheimer’s disease^44,45^. DNA methylation also plays an important role in the pathogenesis of T2DM^45,46^. In order to better understand the etiology of AD and T2DM, we jointly investigated the genetic variants, DNA methylation and gene expression profiles, multiple phenotypes, AD and T2DM using causal inference pipelines. We found that gene *POU3F2* regulated methylations of POU3F2, KIF4B and TMSL3. Alternations in methylation of three genes directly caused the development of AD and T2DM. Furthermore, methylation levels of three genes regulated gene expressions in dopaminergic synapse and AMPK pathways, which in turn caused AD and T2DM via CHL/HDL Ratio (Fig. 4A). Recent advance revealed that alterations of the dopaminergic system contributes to memory and reward dysfunction and the dopaminergic system may well be involved in the occurrence of AD^47,48^. Recent studies also unravel that the brain damage in AD is linked to an over-activation of AMPK, which leads to the loss of the ability of neurons to grow axons and the modification of the tau proteins resulting in tangles of tau^49^. AMPK functions as a key energy sensor. AMPK signaling elicits insulin-sensitizing effects and may be implicated in stimulating glucose up taking in skeletal muscles, fatty acid oxidation in adipose (and other) tissues^50^.

We identified an extremely large number of shared causal paths from genetic variants to both AD and T2DM via DNA methylation, gene expressions and phenotypes. This deep knowledge that uncovered the large number of causal mechanisms of AD and T2DM has profound implications in prevention and treatments of AD and T2DM. This explained why the drugs that were based on inhibition or activation of limited number of paths often failed simply because these limited number of paths cannot cover all causal paths to the diseases. Finally, the empirical evidence that the AD and T2DM shared a large number of causal genes, gene expressions, methylations and pathways supported hypothesis that AD can be considered as “type 3 diabetes”.

## Methods

All methods were carried out in accordance with relevant guidelines and regulations.

### ROSMAP data

The data came from two longitudinal cohort studies of older persons. ROS started in 1994 and enrolled Catholic nuns, priests, and brothers from more than 40 communities across United States, and MAP started in 1997 and enrolled participants with diverse backgrounds and socioeconomic status from continuous care retirement communities throughout northeastern Illinois, as well as from individual homes across the Chicago metropolitan area^19^. These two studies are managed by the same team of investigators. Structured, quantitative neuropathological examinations are performed at a single site. Therefore, the data can be combined for analysis. Multi-layered omics datasets are generated from biospecimens donated by ROS and MAP participants, including genotypes, DNA methylation profiles and RNA-seq. The genotype data were generated by Affymetrix or the Illumina Omniquad express gene chips and were imputed using the 1000 Genomes Project data as reference. DNA methylation profiles were measured using the Illumina Infinium HumanMethylation450 beadset. RNA-seq data were generated using the Illumina HiSeq with 101 bp paired-end reads. Multiple phenotypes including clinical diagnosis, cognitive function, measures of lifestyle, behavior, and activity, chronic medical conditions and risk factors were measured. A total of 432 individuals who simultaneously had genotype, RNA-seq, DNA methylation and some phenotypes were included in analysis. We considered 19 phenotypes and environments, two diseases (AD, T2DM), 299 pathways with RNA-Seq in KEGG pathway database, 20,242 methylation genes with 364,661 CpG sites, and 51, 060 genotyped genes with 5,711,541 SNPs (4,283,876 common SNPs, 1,427,665 rare SNPs). All the data were downloaded from https://www.radc.rush.edu/.

The ROSMAP studies were approved by the Institutional Review Board of Rush University Medical Center. Written informed consent was obtained from all subjects, followed by an Anatomic Gift Act for organ donation.

### General procedures for identifying shared genetic loci underlying AD and T2DM

**Figure 5:**
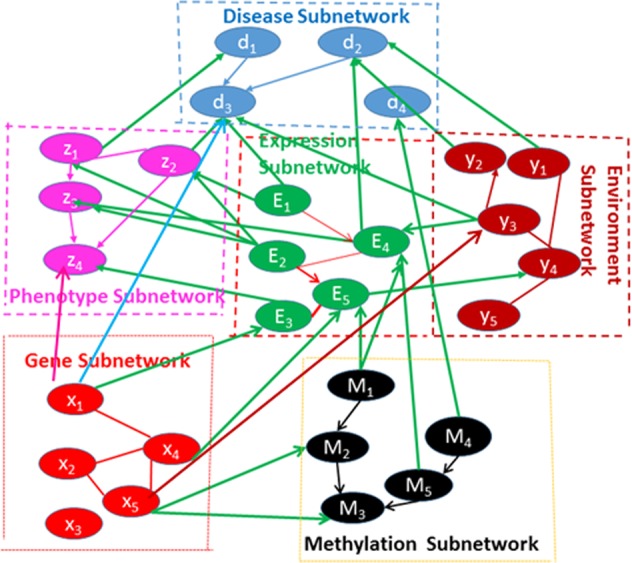
Scheme of multilevel omic networks.

AD and T2DM result from the interplay of DNA sequence variation and nongenetic factors acting through molecular networks^51–53^. Their etiology is complex with many intermediate steps between genetic variation and diseases. Neither traditional GWAS, nor classical multi-omics analysis can identify the causal passes of complex diseases because not all these analyses can identify directed routes from genetic loci to diseases through environments, methylations, gene expressions, and phenotypes. To overcome these limitations, we developed a novel general framework for identifying all possible causal passes from genetic loci to diseases. The framework consists of three steps. The first step is to perform genome-wide causation studies (GWCS) where we test causation of each SNP across the genome to the disease. The additive noise model (ANM) with discrete variants will be used to test for causation^54^ (Methods). We focused on the rare variants in the paper. The second step is to use integer programming (IP) and various modern causal models^55–57^ (Methods) for inferring multilevel genome-wide omics causal networks that integrate genotype subnetworks, environmental subnetworks, methylation subnetworks, gene regulatory subnetworks, intermediate phenotype subnetworks and multiple disease subnetworks into a single connected multilevel genotype-disease network as shown in Fig. 5. The third step is to augment graph theoretical approaches with approximations for developing efficient search algorithms that discover all possible routes starting from the genetic variant node directed to the disease node, including classical Depth First Search (DFS) and Breadth First Search (BFS) algorithms^58–61^.

There are two ways to identify shared dysfunctional genes (SNPs) between AD and T2DM. One way is to use ANM with discrete variables and functional data analysis to conduct genome-wide causation analysis^54,62–64^ for unravelling the direct connections between gene nodes and disease nodes to identify the shared dysfunctional genes between AD and T2DM.

Another way is to search the paths from the gene nodes to AD and T2DM in multilevel causal omics networks.

Association and causation are different concepts. Association between two variables is often characterized by dependence between two variables. Causation is a connection of phenomena where one variable acts or intervenes on another variables and leads to its changes. Therefore, the key component of causation is the generation and determination of values of one variable by another. The mechanism of causation is related to the transference of matter, motion and information. Causation as part of universe connection, is well known that nature consists of autonomous and independent causal generating process modules. These modules will not influence each other^63,65^. In other words, while output of one module may inform or influence input of another module, the events between modules are independent. In the probabilistic language, mechanism is often represented by conditional distribution. Independent mechanism states that “the conditional distribution of each variable given its causes (i.e., its mechanism) does not inform or influence the other conditional distributions”^63^. In GWCS, we only consider two variables. In this case, independence of cause and mechanism (ICM) indicates that the conditional distribution of the effect given its cause is independent of distribution of cause. Consider the genetic analysis of alleles (*A*) with a disease allele *A* a normal allele *a* and with the disease (*D*) (disease *D* and normal *d*). The joint density function $P(a,d)$ can be decomposed into

$$\begin{array}{c}P(A,D)=P(A)P(D|A)\\ \,\,\,\,\,=\,P(D)P(A|D).\end{array}$$

In the association analysis, we assess whether *A* is independent of *D* or not. The relationship between *A* and *D* is symmetric. However, in causal analysis, causations $A\to D$ and $D\to A$ are different. They are asymmetric. Assessing causation is to consider the effect of intervention. Causation $A\to D$ indicates that the effect of *A* is to give rise to disease. However, disease status *D* will not generate allele *A*. Suppose that locus *A* is disease locus and $\,A\to D$. If we change the allele *a* to allele *A*, then we assume that biological mechanism $P(D|A)$ responsible for giving rise to disease. This would hold true independent of the distribution (frequencies)_of allele *A*. If the locus A is disease locus, we can find that the distributions (frequencies) of allele *A* in two different populations are different, but the mechanism $P(D|A)$ would apply in two populations. The conditional probability $P(D|A)$ can also be viewed as penetrance of the allele. The marginal distribution $P(A)$ and conditional distribution $P(D|A)$ contain no information about each other. Both continuous and discrete ANMs satisfy the ICM and will be used for GWCS., The proposed method for genome-wide causation analysis and inferring multilevel causal genotype-methylation-expression-phenotype-disease network was applied to the ROSMAP dataset^19^ with 432 individuals, 19 phenotypes and environments, two diseases (AD, T2DM), 299 pathways with RNA-Seq in KEGG pathway database, 20,242 methylation genes with 364,661 CpG sites, and 51, 060 genotyped genes with 5,711,541 SNPs (4,283,876 common snp, 1,427,665 rare snp) (imputed by 1000 Gnome Data). The inferred genotype-expression-methylation-phenotype-disease network consisted of 2,814 nodes and 22,184 edges where the edges were presented in the network if the path coefficients were significantly from zero with P-values < 0.05.

There were two ways to connect a gene (or SNP) to AD (T2DM). If a gene (or SNP) showed causation to AD (T2DM) by statistical causal test, then the gene (SNP) was directly connected to AD (T2DM) in the causal network. Such gene (SNP) was called AD (T2DM) directly connected gene (SNP). We may observe the connection between a gene (SNP) and AD (T2DM) via multiple edges (paths) in the constructed multilevel causal network. Then, the gene (SNP) that was indirectly connected to AD (T2DM) via paths in the multilevel causal network was called AD (T2DM) indirectly connected gene (SNP).

### Genome-wide causation studies

Unlike GWAS where we test the association of each variant across the genome with the disease, genome-wide causation studies (GWCS) test the causation of each variant across the genome to the disease. Additive noise models (ANMs) with discrete variables will be used for GWCS^54,62–64^. The procedures that use the ANMs for GWCS are summarized as follows^14,54,63^.

### Procedures for causal genetic analysis using ANM

1. Fit the following nonlinear integer regression to the data.
  $$Y=f(X)+{N}_{Y}.$$
  Calculate the residuals ${\hat{N}}_{Y}=Y-\hat{f}(X)$.
2. Fit the following nonlinear integer regression to the data.
  $$X=g(Y)+{N}_{X}.$$
  Calculate the residuals ${\hat{N}}_{X}=X-\hat{g}(Y)$.
3. Test for independence.

The contingence table and Fisher’s exact test can be used to test independence. Let the statistic for testing the independence between ${\hat{N}}_{Y}$ and *X* as ${\Delta }_{X\to Y}$ and the statistic for testing the independence between ${\hat{N}}_{X}$ and *Y* as ${\Delta }_{Y\to X}$.

The null hypothesis for testing the causation of the variant is

*H*_0_: no causation between variables *X* and *Y*.

The statistic for testing the causation between two *X* and *Y* is defined as

$${T}_{C}=|{\Delta }_{X\to Y}-{\Delta }_{Y\to X}|.$$

When ${T}_{C}$ is large, the causation between genetic variant $X$ and disease status $Y$ exists. When ${T}_{C}\approx 0$, this indicates that no causal decision can be made. Since the distribution of the test statistic ${T}_{C}$ is difficult to calculate, P-value for testing the causation of the variant $X$ can be calculated by permutations.

To improve the performance of causation analysis of rare variants, we first calculate the functional principle component score (FPCS) of the rare variants within a gene^64^ to summerize information of all rare variants within the gene. Then, the continuous FPCS are discreterized. Finally, the ANMs with discrete variables can be used to test causation of discreterized FPCS with the disease.

### Structural equations for construction of causal networks

Directed graphical models and structural equations can be used as a tool to model the complex causal structures among variables^64,66^. A graphical model consists of nodes and edges. The nodes represent variables and edges represent the dependence structures among variables. A directed graphic model is defined as the graph in which all the inter-node connections have a direction visually denoted by an arrowhead. Directed acyclic graphics (DAGs) are defined as directed graphics with no cycles. In other words, we can never start at a node *X*, travel edges in the directions of the arrows and get back to the node *X*. A DAG with nodes encodes conditional dependence structure of the variables ${Y}_{1},\ldots {Y}_{n}$. We define the parents of a node as the nodes pointing directly to it. The concept of parents provides an easy way to read off conditional independence from DAGs.

Traditional regressions describe one-way or unidirectional relationships among variables in which the variables on the left sides of the equations are dependent variables and the variables on the right sides of the equations are explanatory variables or independent variables. The explanatory variables are used to predict the outcomes of the dependent variables. However, in many cases, there are two ways, or simultaneous relationships between the variables. Variables in some equations are response variables, but will be predictors in other equations. The variables in equations may influence each other. It is difficult to distinguish dependent variables and explanatory variables. The structural equation models (SEMs) are a powerful mathematic tool to describe such data generating mechanism and infer causal relationships among the variables.

The SEMs classify variables into two class variables: endogenous and exogenous variables. The jointly dependent variables that are determined in the model are called endogenous variables. The explanatory variables that are determined outside the model or predetermined are called exogenous variables. In the genotype-phenotype networks, the phenotype variables such as BMI, cognitive function, working memory, are endogenous variables, age, sex, race, environments and genotypes are exogenous variables. In the genotype-expression networks, the gene expressions are endogenous variables and genotypes are exogenous variables. In the methylation-expression networks, gene expressions are endogenous variables and methylations are exogenous variables.

We consider *M* endogenous variables. Assume that *n* individuals are sampled. We denote the *n* observations on the *M* endogenous variables by the matrix $Y=[{y}_{1},{y}_{2},\ldots ,{y}_{M}]$, where ${y}_{i}={[{y}_{1i},{y}_{2i},\ldots ,{y}_{ni}]}^{T}$ is a vector of collecting *n* observation of the endogenous variable *i*. Exogenous variables are denoted by $X=[{x}_{1},{x}_{2},\ldots ,{x}_{M}]$ where ${x}_{i}={[{x}_{1i},{x}_{2i},\ldots ,{x}_{ni}]}^{T}$. Similarly, random errors are denoted by E $=\,[{e}_{1},{e}_{2},\ldots ,{e}_{M}]$ where we assume $E[{e}_{1}]=0$ and $E[{e}_{i}{{e}_{i}}^{T}]={\sigma }_{i}^{2}{I}_{n}$ for $i=1\ldots M$ The linear structural equations for modeling relationships among variables can be written as:

1 $$\begin{array}{c}{y}_{1}{\gamma }_{11}+{y}_{2}{\gamma }_{21}+\ldots +{y}_{M}{\gamma }_{M1}+{x}_{1}{\beta }_{11}+{x}_{2}{\beta }_{21}+\ldots +{x}_{K}{\beta }_{K1}+{e}_{1}=0\\ \vdots \,\,\,\,\,\,\,\,\,\,\,\vdots \,\,\,\,\,\,\,\,\vdots \\ {y}_{1}{\gamma }_{1M}+{y}_{2}{\gamma }_{2M}+\ldots +{y}_{M}{\gamma }_{MM}+{x}_{1}{\beta }_{1M}+{x}_{2}{\beta }_{2M}+\ldots +{x}_{K}{\beta }_{KM}+{e}_{M}=0\end{array}$$

where the *γ*’s and *β*’s are the structural parameters of the system that are unknown. Variables in the SEMs can be classified into two basic types of variables: observed variables that can be measured and the residual error variables that cannot be measured and represent all other unmodeled causes of the variables. Most observed variables are random. Some observed variables may be nonrandom or control variables (e.g. genotypes, drug dosages) whose values remain the same in repeated random sampling or might be manipulated by the experimenter. The observed variables will be further classified into exogenous variables, which lie outside the model, and endogenous variables, whose values are determined through joint interaction with other variables within the system. All nonrandom variables can be viewed as exogenous variables. The terms exogenous and endogenous are model specific. It may be that an exogenous variable in one model is endogenous in another.

Traditionally, we often select one endogenous variable to appear on the left-hand side of the equation. Specifically, the i-th equation is

2 $${y}_{i}={y}_{1}{\gamma }_{1i}+\ldots +{y}_{i-1}{\gamma }_{i-1i}+{y}_{i+1}{\gamma }_{i+1i}+\ldots +{y}_{M}{\gamma }_{Mi}+{x}_{1}{\beta }_{1i}+\ldots +{x}_{K}{\beta }_{Ki}+{e}_{i},$$

where ${\gamma }_{ji}$ is a path coefficient that measures the strength of the causal relationship from ${Y}_{j}$ to ${y}_{i}$, ${\beta }_{ki}$ is a path coefficient from the exogenous variable to the endogenous variable which measure the causal effect of the exogenous variable ${x}_{k}\,$ on the endogenous variable ${y}_{i}$. The coefficients ${\gamma }_{ji}=0\,$ and ${\beta }_{ki}=0$ imply the zero direct influence of ${Y}_{j}$ and ${x}_{k}$ on ${Y}_{i}$, respectively and are usually omitted from the equation. Therefore, Eq. (2) is reduced to

3 $${y}_{i}={Y}_{-i}{\gamma }_{i}+{X}_{i}{\beta }_{i}+{e}_{i}={W}_{i}{\Delta }_{i}+{e}_{i}$$

where ${Y}_{-i}$ is a vector of the endogenous variables after removing variable ${Y}_{i}$, ${\gamma }_{i}$ is a vector of the path coefficients associated with ${Y}_{-i}$, and

$${W}_{i}=[{Y}_{-i},\,{X}_{i}],\,{\Delta }_{i}={[{\gamma }_{i},{\beta }_{i}]}^{T}.$$

Multiplying by the matrix ${X}^{T}$ on both sides of Eq. (3), we obtained

4 $${X}^{T}{y}_{i}={X}^{T}{W}_{i}{\Delta }_{i}+{X}^{T}{e}_{i}.$$

Estimation of the parameters in the structural equations is rather complex. It involves many different estimation methods with varying statistical properties. We used two stage least squares (2SLS) method to estimate the parameters. In general, the causal networks are sparse. Using weighted least square and *l*_1_-norm penalization of Eq. (4), we can form the following optimization problem to estimate the structure of causal network:

$$\mathop{{\rm{\min }}}\limits_{{\Delta }_{i}}f({\Delta }_{i})+\lambda ||{\Delta }_{i}|{|}_{1}$$

Where

5 $$f({\Delta }_{i})={({X}^{T}{y}_{i}-{X}^{T}{W}_{i}{\Delta }_{i})}^{T}{({X}^{T}X)}^{-1}({X}^{T}{y}_{i}-{X}^{T}{W}_{i}{\Delta }_{i}).$$

The alternating direction method of multipliers (ADMM) and proximal methods can be used to estimate the parameters and structure of causal network^64,67,68^.

### Functional structural equation models for construction of gene-based causal networks

The SEMs carry out variant by variant analysis. However, the power of the traditional variant-by-variant analytical tools for construction of causal networks with rare variants as exogenous variables will be limited. Large simulations have shown that combining information across multiple variants in a genomic region of analysis will greatly enhance the power to infer causal networks with rare variants as exogenous variables. To utilize multi-locus genetic information, we propose to use a genomic region or a gene as a unit in construction of causal networks and develop sparse structural functional equation models (SFEMs) for causal network analysis^66^.

We define a genotype function. Let *t* be a genomic position. Define a genotype function ${x}_{i}(t)$ of the *i*-th individual as

$${x}_{i}(t)=\{\begin{array}{c}2{P}_{q}(t)\,\\ {P}_{q}\,({\rm{t}})-{P}_{Q}(t)\,\\ -2{P}_{Q}(t)\end{array}\begin{array}{c}QQ\\ Qq\\ qq\end{array}$$

where *Q* and *q* are two alleles of the marker at the genomic position *t*, ${P}_{Q}({\rm{t}})\,$ and ${P}_{q}({\rm{t}})$ are the frequencies of the alleles *Q* and *q*, respectively. Suppose that we are interested in *k* genomic regions or genes $[{a}_{j},\,{b}_{j}]$ denoted as ${T}_{j},\,j=1,\ldots ,K$. We consider the following functional structural equation models (FSEMs):

6 $$\begin{array}{c}{y}_{1}{\gamma }_{11}+{y}_{2}{\gamma }_{21}+\ldots +{y}_{M}{\gamma }_{M1}+{\int }_{{T}_{1}}{x}_{1}(t){\beta }_{11}(t)dt+\ldots +{\int }_{{T}_{k}}{x}_{k}(t){\beta }_{k1}(t)dt+{e}_{1}=0\\ \vdots \,\,\,\,\,\,\,\,\,\,\,\vdots \,\,\,\,\,\,\,\,\vdots \\ {y}_{1}{\gamma }_{1M}+{y}_{2}{\gamma }_{2M}+\ldots +{y}_{M}{\gamma }_{MM}+{\int }_{{T}_{1}}{x}_{1}(t){\beta }_{1M}(t)dt+\ldots +{\int }_{{T}_{k}}{x}_{k}(t){\beta }_{kM}(t)dt+{e}_{M}=0\end{array}$$

where ${\beta }_{ij}(t),\,j=1,\ldots ,k,\,i=1,\ldots ,M$ are genetic effect functions.

Functional principal components (FPCs) are efficient summary statistics. The FPCs simultaneously employs genetic information of the individual variants and correlation information (LD) among all variants. For each genomic region or gene, we use functional principal component analysis to calculate principal component function. Let *N* be the number of sampled individuals. We expand ${x}_{nj}(t),\,n=1,\ldots ,N,\,j=1,\ldots ,k$ in each genomic region in terms of orthogonal principal component functions:

$${x}_{nj}(t)=\,\mathop{\sum }\limits_{l=1}^{{L}_{j}}{\eta }_{njl}{\phi }_{jl}(t),\,j=1,\ldots ,k,$$

where ${\phi }_{jl}(t),\,j=1,\ldots ,k,\,l=1,\ldots ,{L}_{j}$ are the *l*-th principal component function in the *j*-th genomic region or gene and ${\eta }_{njl}$ are the functional principal component scores of the *n*-th individual. Using the functional principal component expansion of ${x}_{nj}(t)$, we can transform the FSEMs (6) into the traditional multivariate SEMs (1).

### Integer programming for causal network learning

Given the dataset, learning causal networks is the task of finding network structures that best fits the data^57,64^. We used “score and search” methods to learn causal networks via maximizing the score metrics that characterize the causal networks. The “score and search” algorithms consist of two parts: (1) formulate objective function (global score for the whole network) using the score function for each node and (2) search algorithm.

We collected all nodes with directed edges in the causal network into a DAG, denoted as $G=(V,E)$. The score (objective function) for the DAG *G* was defined as

$$Score\,(G)=\sum _{j\in V}Scor{e}_{j}(G)m$$

where $Scor{e}_{j}(G)$ was a score for the node *j* in the network. The $Scor{e}_{j}(G)$ was calculated as $f({\Delta }_{j})$ via solving the optimization problem (5). Therefore, the total score can be decomposed into a sum of score for all nodes in the DAG. In addition, the $Scor{e}_{j}(G)$ is entirely determined by the parent set of the node *j* in *G*. A DAG can be encoded by the set $W=\{{W}_{1},\ldots ,{W}_{p}\}$ of parent variables for all nodes *V* in the graph *G*. We use $C(j,{W}_{j})$ to denote a score function for the pair of node *j* and its parent set ${W}_{j}$. Therefore, the total score for the DAG *G* was given by

$$C(D)=\sum _{i\in V}C(v,{W}_{v}).$$

The learning task is to find a DAG that optimizes the global score *C(D)* over all possible DAGs *D* or parent sets^57^:

$$\mathop{\min }\limits_{D}\sum _{i\in V,\,{W}_{v}\in D}C(v,{W}_{v}).$$

Integer linear programming (ILP) was used as a search algorithm^57^. A DAG learning was formulated as the ILP as follows. We define a variable $x({W}_{v}\to v)$ to indicate the presence or absence of the parent set ${W}_{v}$ in the DAG. In other words, $x({W}_{v}\to v)=1$ if and only if it is the parent set for the node *v*. The parent set ${W}_{v}$ can be an empty set. The objective function for the ILP formulation of a DAG learning can be defined as

7 $$\mathop{\sum }\limits_{v=1}^{P}\mathop{\sum }\limits_{{j}_{v}=1}^{{J}_{v}}C(v,{W}_{{j}_{v}})x({W}_{{j}_{v}}\to v).$$

The goal was to find a candidate parent set ${W}_{v}$ for each node $v$ by optimizing the objective function in (7). It is clear that every DAG can be encoded by a zero-one indicator variable. However, any set of zero-one numbers may not encode a DAG. A set of linear constraints must be posted to make the set of indicator variables to represent a DAG. Without constraints all indicator variables for the parent sets will be equal to either zero or one. These solutions will not form a DAG. The constraints need to be imposed to ensure that the solutions encode a DAG. This constraint that is referred to as convexity constraint, can be expressed as

8 $$\mathop{\sum }\limits_{{i}_{j}=1}^{{I}_{j}}x({W}_{{i}_{j}}\to j)=1,\,j=1,\ldots ,p$$

The convexity constraints (8) can define a directed graph. However, the generated directed graph may have cycles. To eliminate a cycle, we need to impose the following constraint to ensure that any subset *C* of the nodes *V* in a DAG must contain at least one node that has no parent in the subset *C*

9 $$\forall \,C\subseteq \sum _{j\in C}\sum _{W:W{\cap }^{}C=\varnothing }x(W\to j)\ge 1,$$

which is referred to as cluster-based constraints. Our goal is to find a candidate parent set *W*_*j*_ for each node *j* by optimizing objective function (7) subject to the constraints (8) and (9).

The branch and bound method is a popular algorithm ensured to find an optimal solution to the 0–1 ILP problem^57^. Let the LP solution represent “solution of the current linear relaxation”. The basic idea of the branch and bound method is to successively divide the ILP problem into smaller problems that are easy to solve and reduce the search space. Briefly, the branch and bound algorithm is summarized as follows. Step 1: Let $\hat{x}$ be the LP solution. Step 2: if there are, valid constraints not satisfied by $\hat{x}$ add them and go to Step 1; otherwise if the solution $\hat{x}$ is an integer then stop, the current problem is solved; otherwise branch on a variable with a non-integer part in $\hat{x}$ to generate two new sub-IP problems. We then again use branch and bound algorithms to solve two sub-ILP problems^57^.

### Multilevel causal networks

Multilevel causal omics networks integrated genotype subnetworks, methylation subnetworks, gene expression subnetworks, the intermediate phenotype subnetworks and multiple disease subnetworks into a single connected multilevel genotype-disease networks to reveal the deep causal chain of mechanisms underlying the diseases^64^. ILP was extended from a single causal network estimation to joint multiple causal network estimations to integrate genomic, epigenomic and phenotype data.

For the convenience of discussion, consider *M* gene expression variables ${Y}_{1},\ldots ,{Y}_{M},$ *Q* methylation variables ${Z}_{1},\ldots ,{Z}_{Q}$, and *K* genotype variables ${X}_{1},\ldots ,{X}_{K}$. Let $p{a}_{D}(D)\,$ be the parent set of the node *d* including gene expression, methylation and genotype variables. Consider three types of SEMs. First, we consider a general SEM model for the gene expression:

10 $${Y}_{d}=\sum _{i\in p{a}_{D}(D)}{f}_{di}({Y}_{i})+\sum _{q\in p{a}_{D}(D)}{f}_{dq}({Z}_{q})+\sum _{j\in p{a}_{D}(D)}{f}_{dj}({X}_{j})+{\varepsilon }_{d},\,d=1,\ldots M$$

And

11 $${Z}_{q}=\sum _{l\in p{a}_{Q}(q)}{f}_{ql}({Z}_{l})+\sum _{m\in p{a}_{Q}(q)}{f}_{qm}({X}_{m})+{\varepsilon }_{q},\,q=1,\ldots Q$$

where ${f}_{d}$ and ${f}_{q}$ are linear functions from ${R}^{|p{a}_{D}|}\to R$ and ${R}^{|p{a}_{Q}|}\to R$, respectively, and the errors ${\varepsilon }_{d}$ and ${\varepsilon }_{q}$ are independent, following distributions ${P}_{{\varepsilon }_{d}}$ and ${P}_{{\varepsilon }_{Q}}$, respectively. Equation (10) define a causal network that connects gene expressions, methylations and genotypes. Equation (11) define a causal network that connects methylations and genotypes.

### Integer programming as a general framework for joint estimation of multiple causal networks

We collected multiple types of data: genotype, gene expression, methylation, and phenotype and disease data. We wanted to estimate multiple causal networks with different types of data^64^.

The scores of the nodes ${Y}_{d}$ and ${Z}_{q}$ were, respectively, given by

12 $$C({Y}_{d},{W}_{di})={Y}_{d}^{T}(I-{D}_{Y}^{i}{({({D}_{Y}^{i})}^{T}{D}_{Y}^{i})}^{-1}{({D}_{Y}^{i})}^{T}){Y}_{d}$$

and

13 $$C({Z}_{q},{W}_{ql})={Z}_{q}^{T}(I-{D}_{Z}^{l}{({({D}_{Z}^{l})}^{T}{D}_{Z}^{l})}^{-1}{({D}_{Z}^{l})}^{T}){Z}_{q}$$

where matrices ${D}_{Y}^{i}$ and ${D}_{Z}^{l}$ corresponded to the parent sets ${W}_{di}$ and ${W}_{ql}$.

Let ${V}_{E}$ be the set of nodes in the gene expression network and ${V}_{M}$ be the set of nodes in the methylation network. Let ${C}_{E}$ be a subset of nodes in ${V}_{E}$ and ${C}_{M}$ be a subset of nodes in ${V}_{M}$. A joint expression and methylation causal network can be formulated as the following ILP:

14 $$\begin{array}{c}\min \,\mathop{\sum }\limits_{d=1}^{M}\sum _{i\in p{a}_{D}(d)}C(d,{W}_{di})x({W}_{di}\to d)+\mathop{\sum }\limits_{q=1}^{Q}\sum _{l\in p{a}_{Q}(q)}C(q,{W}_{ql})x({W}_{ql}\to q)\\ {\rm{s}}{\rm{.t}}.\,\,\sum _{i\in p{a}_{D}(d)}x({W}_{di}\to d)=1,\,d=1,\ldots ,M,\,\\ \sum _{i\in p{a}_{Q}(q)}x({W}_{ql}\to q)=1,\,q=1,\ldots ,Q,\\ \forall \,{C}_{E}\subseteq {V}_{E}:\sum _{d\in {C}_{E}}\sum _{{W}_{d}:{W}_{d}{\cap }^{}{C}_{E}=\varnothing }x({W}_{d}\to d)\ge 1,\\ \forall \,{C}_{M}\subseteq {V}_{M}:\sum _{q\in {C}_{M}}\sum _{{W}_{q}:{W}_{q}{\cap }^{}{C}_{M}=\varnothing }x({W}_{q}\to q)\ge 1.\end{array}$$

Using branch and bound and other methods for solving the ILP, we can solve the ILP problem (14) to obtain the best joint causal genotype-methylation-expression and genotype-methylation network fitting the data.

### Summary statistics for representation of groups of gene expressions

Generalized low rank models were used to segment (cluster) the data. Principal component analysis (PCA) was used to reduce data dimensions. The PCs were used to summarize the gene expression data in pathways and clusters^69^.

### Simulations of causal networks

We simulated causal networks with genes (genotype) and gene expressions as the nodes of the networks. We randomly selected 8 genes (30 node model) and 10 genes (50 node model) from the ROSMAP dataset. The genotype information of multiple SNPs within a gene was summarized by FPCA scores which were taken as the values of the gene node. We used R package PCALG^70,71^ to randomly generate DAG with 30 nodes (edges ranging from 70 to 90), and with 50 noes (edges ranging from 80 to 110). The values of the gene expression nodes were generated by the following model^66,70–72^

$${y}_{i}=\sum _{j\in pa({y}_{i})}{\gamma }_{ji}{y}_{j}+\sum _{k\in pa({y}_{i})}{\beta }_{ki}{x}_{k}+{e}_{i},\,i=1,\ldots ,M,$$

where $pa({y}_{i})$ is the set of parents of the node ${y}_{i}$, the coefficients ${\gamma }_{ji}$ and ${\beta }_{ki}$ followed a uniform distribution $u(1,2)$, ${e}_{i}$ followed a normal distribution $N(0,1)$.

A total of 100, 300, 500 and 1,000 DAGs were generated. The number of replication was 1,000. Let ${N}_{t}$ be the total number of edges among simulated DAGs, ${N}_{0}$ the total number of edges that were not presented in the simulated DAGs, ${N}_{True}$ the total number of edges detected by the algorithm and ${N}_{False}$ the false edges directed among ${N}_{0}$. Then the false discovery rate (FDR) was defined as $\frac{{N}_{False}}{{N}_{0}}$ and power of detection defined as $\frac{{N}_{True}}{{N}_{t}}$.

### Ethical approval and informed consent

The ROSMAP studies were approved by the Institutional Review Board of Rush University Medical Center. Written informed consent was obtained from all subjects, followed by an Anatomic Gift Act for organ donation.

## Supplementary information

Supplementary information.

## Data Availability

All data are publically available and can be downloaded from RADC Research Resource Sharing Hub (https://www.radc.rush.edu/).

## References

[1] Zhuang, Q.-S., Zheng, H., Gu, X.-D., Shen, L. & Ji, H.-F. Detecting the genetic link between Alzheimer’s disease and obesity using bioinformatics analysis of GWAS data. *Oncotarget* **8** (2017). 10.18632/oncotarget.19115 PMC5593533 28915562

[2] Song MK, Bischoff DS, Song AM, Uyemura K, Yamaguchi DT (2017). Metabolic relationship between diabetes and Alzheimers Disease affected by Cyclo(His-Pro) plus zinc treatment. BBA Clinical.

[3] Lashley T (2018). Molecular biomarkers of Alzheimers disease: progress and prospects. Disease Models & Mechanisms.

[4] Fischer R, Maier O (2015). Interrelation of Oxidative Stress and Inflammation in Neurodegenerative Disease: Role of TNF. Oxidative Medicine and Cellular Longevity.

[5] Li, X., Leng, S. & Song, D. Link between type 2 diabetes and Alzheimer’s disease: from epidemiology to mechanism and treatment. *Clinical Interventions in Aging* **549**. 10.2147/cia.s74042 (2015). 10.2147/CIA.S74042 PMC4360697 25792818

[6] Baglietto-Vargas D, Shi J, Yaeger DM, Ager R, Laferla FM (2016). Diabetes and Alzheimer’s disease crosstalk. Neuroscience & Biobehavioral Reviews.

[7] Pugazhenthi S, Qin L, Reddy PH (2017). Common neurodegenerative pathways in obesity, diabetes, and Alzheimers disease. Biochimica et Biophysica Acta (BBA). Molecular Basis of Disease.

[8] Akter K (2011). Diabetes mellitus and Alzheimers disease: shared pathology and treatment?. British Journal of Clinical Pharmacology.

[9] Arvanitakis Z, Wilson RS, Bienias JL, Evans DA, Bennett DA (2004). Diabetes Mellitus and Risk of Alzheimer Disease and Decline in Cognitive Function. Archives of Neurology.

[10] Arvanitakis Z (2006). Diabetes is related to cerebral infarction but not to AD pathology in older persons. Neurology.

[11] Talbot K (2012). Demonstrated brain insulin resistance in Alzheimer’s disease patients is associated with IGF-1 resistance, IRS-1 dysregulation, and cognitive decline. Journal of Clinical Investigation.

[12] Hohman TJ (2015). GSK3β Interactions with Amyloid Genes: An Autopsy Verification and Extension. Neurotoxicity Research.

[13] Karki R, Kodamullil AT, Hofmann-Apitius M (2017). Comorbidity Analysis between Alzheimer’s Disease and Type 2 Diabetes Mellitus (T2DM) Based on Shared Pathways and the Role of T2DM Drugs. Journal of Alzheimers Disease.

[14] Jiao, R. *et al*. Bivariate Causal Discovery and Its Applications to Gene Expression and Imaging Data Analysis. *Frontiers in Genetics* **9** (2018). 10.3389/fgene.2018.00347 PMC6127271 30233639

[15] Bennett DA, Schneider JA, Arvanitakis Z, Wilson RS (2012). Overview and Findings from the Religious Orders Study. Current Alzheimer Research.

[16] Bennett DA (2012). Overview and Findings from the Rush Memory and Aging Project. Current Alzheimer Research.

[17] Kandel ER (2012). The molecular biology of memory: cAMP, PKA, CRE, CREB-1, CREB-2, and CPEB. Molecular Brain.

[18] Saura, C. A. & Valero, J. The role of CREB signaling in Alzheimer’s disease and other cognitive disorders*. Reviews in the Neurosciences* **22** (2011). 10.1515/RNS.2011.018 21476939

[19] White Charles C., Yang Hyun-Sik, Yu Lei, Chibnik Lori B., Dawe Robert J., Yang Jingyun, Klein Hans-Ulrich, Felsky Daniel, Ramos-Miguel Alfredo, Arfanakis Konstantinos, Honer William G., Sperling Reisa A., Schneider Julie A., Bennett David A., De Jager Philip L. (2017). Identification of genes associated with dissociation of cognitive performance and neuropathological burden: Multistep analysis of genetic, epigenetic, and transcriptional data. PLOS Medicine.

[20] Cerasuolo, J. & Izzo, A. Persistent impairment in working memory following severe hyperglycemia in newly diagnosed type 2 diabetes. *Endocrinology, Diabetes & Metabolism Case Reports* **2017** (2017). 10.1530/EDM-17-0101 PMC5744618 29302328

[21] Huang R-R (2015). Spatial working memory impairment in primary onset middle-age type 2 diabetes mellitus: An ethology and BOLD-fMRI study. Journal of Magnetic Resonance Imaging.

[22] Montoya, J. C. *et al*. Global differential expression of genes located in the Down Syndrome Critical Region in normal human brain. *Colombia**Medica* 154–161. 10.25100/cm.v45i4.1640 (2014). PMC4350380 25767303

[23] Kohli, M. A. *et al*. Segregation of a rare TTC3 variant in an extended family with late-onset Alzheimer disease. *Neurology**Genetics* **2** (2016). 10.1212/NXG.0000000000000041 PMC4817909 27066578

[24] Berto G (2007). The Down syndrome critical region protein TTC3 inhibits neuronal differentiation via RhoA and Citron kinase. Journal of Cell Science.

[25] Galaria II, Nicholl SM, Roztocil E, Davies M (2004). Differential Regulation Of Erk1/2 And P38Mapk By Components Of The Rho Signaling Pathway During Sphingosine-1-Phosphate (Sip) - Induced Smooth Muscle Cell (Smc) Migration. Cardiovascular Pathology.

[26] Maiese K (2018). Forkhead Transcription Factors: Formulating a FOXO Target for Cognitive Loss. Current Neurovascular Research.

[27] Kim, J. H., Choi, J. S. & Lee, B. H. PI3K/Akt and MAPK pathways evoke activation of FoxO transcription factor to undergo neuronal apoptosis in brain of the silkworm Bombyx mori (Lepidoptera: Bombycidae). Cell Mol Biol (Noisy-le-grand). Suppl.58:OL1780–1785 (2012). 23046871

[28] Zhang Z (2016). Insulin-Dependent Regulation of mTORC2-Akt-FoxO Suppresses TLR4 Signaling in Human Leukocytes: Relevance to Type 2 Diabetes. Diabetes.

[29] Yin X (2017). Association of PI3K/AKT/mTOR pathway genetic variants with type 2 diabetes mellitus in Chinese. Diabetes Research and Clinical Practice.

[30] Kitagishi Y (2014). Certain Diet and Lifestyle May Contribute to Islet β-cells Protection in Type-2 Diabetes via the Modulation of Cellular PI3K/AKT Pathway. The Open Biochemistry Journal.

[31] Mohammad Ahmadi Soleimani S., Ekhtiari Hamed, Cadet Jean Lud (2016). Drug-induced neurotoxicity in addiction medicine. Progress in Brain Research.

[32] Yang S-P (2015). Risk of type 2 diabetes mellitus in female breast cancer patients treated with morphine: A retrospective population-based time-dependent cohort study. Diabetes Research and Clinical Practice.

[33] Trucco, M. Genetic and Environmental Pathways in Type 1 Diabetes Complications. 10.21236/ada544029 (2009).

[34] Jager, C. A. D. & Kovatcheva, A. Summary and discussion: Methodologies to assess long-term effects of nutrition on brain function. *Nutrition Reviews* **68** (2010). 10.1111/j.1753-4887.2010.00332.x 20946369

[35] Hooper C, De Souto Barreto P, Pahor M, Weiner M, Vellas B (2018). The Relationship of Omega 3 Polyunsaturated Fatty Acids in Red Blood Cell Membranes with Cognitive Function and Brain Structure: A Review Focussed on Alzheimer’s Disease. J. Prev. Alzheimers Dis..

[36] Grimm MOW, Michaelson DM, Hartmann T (2017). Omega-3 fatty acids, lipids, and apoE lipidation in Alzheimer’s disease: a rationale for multi-nutrient dementia prevention. Journal of Lipid Research.

[37] Ramalho RM, Viana RJ, Low WC, Steer CJ, Rodrigues CM (2008). Bile acids and apoptosis modulation: an emerging role in experimental Alzheimers disease. Trends in Molecular Medicine.

[38] Pan X (2017). Metabolomic Profiling of Bile Acids in Clinical and Experimental Samples of Alzheimer’s Disease. Metabolites.

[39] Bouchouirab, F.-Z., Fortin, M., Noll, C., Dubé, J. & Carpentier, A. C. Plasma Palmitoyl-Carnitine (AC16:0) Is a Marker of Increased Postprandial Nonesterified Incomplete Fatty Acid Oxidation Rate in Adults With Type 2 Diabetes. *Canadian**Journal of Diabetes* **42** (2018). 10.1016/j.jcjd.2017.09.002 29129455

[40] Wang, S. *et al*. Plasma bile acid changes in type 2 diabetes correlated with insulin secretion in two-step hyperglycemic clamp. *Journal of Diabetes*. 10.1111/1753-0407.12771 (2018). 10.1111/1753-0407.12771 29664215

[41] Chávez-Talavera, O., Tailleux, A., Lefebvre, P. & Staels, B. Bile Acid Control of Metabolism and Inflammation in Obesity, Type 2 Diabetes, Dyslipidemia, and Nonalcoholic Fatty Liver Disease. *Gastroenterology* **152** (2017). 10.1053/j.gastro.2017.01.055 28214524

[42] Grimm, M. O. W., Mett, J., Grimm, H. S. & Hartmann, T. APP Function and Lipids: A Bidirectional Link. *Frontiers in Molecular Neuroscience* **10** (2017). 10.3389/fnmol.2017.00063 PMC5344993 28344547

[43] Huang Y-T, Iwamoto K, Kurosaki T, Nasu M, Ueda S (2005). The neuronal POU transcription factor Brn-2 interacts with Jab1, a gene involved in the onset of neurodegenerative diseases. Neuroscience Letters.

[44] Nagata K, Mano T, Murayama S, Saido TC, Iwata A (2018). DNA methylation level of the neprilysin promoter in Alzheimers disease brains. Neuroscience Letters.

[45] Shen, J. & Zhu, B. Integrated analysis of the gene expression profile and DNA methylation profile of obese patients with type 2 diabetes. *Molecular Medicine Reports*. 10.3892/mmr.2018.8804 (2018). 10.3892/mmr.2018.8804 PMC5983955 29620215

[46] Elliott HR (2017). Role of DNA Methylation in Type 2 Diabetes Etiology: Using Genotype as a Causal Anchor. Diabetes.

[47] Martorana, A. & Koch, G. Is dopamine involved in Alzheimers disease?. *Frontiers in Aging* Neuroscience **6** (2014). 10.3389/fnagi.2014.00252 PMC4174765 25309431

[48] Nobili, A. *et al*. Dopamine neuronal loss contributes to memory and reward dysfunction in a model of Alzheimer’s disease. *Nature**Communications* **8** (2017). 10.1038/ncomms14727 PMC5382255 28367951

[49] Domise Manon, Vingtdeux Valérie (2016). AMPK in Neurodegenerative Diseases. Experientia Supplementum.

[50] Saha, A., Coughlan, K., Valentine, R. & Ruderman, N. AMPK activation: a therapeutic target for type 2 diabetes? *Diabetes, Metabolic Syndrome and Obesity: Targets and Therapy* **241**. 10.2147/dmso.s43731 (2014). 10.2147/DMSO.S43731 PMC4075959 25018645

[51] Huan T (2015). Integrative network analysis reveals molecular mechanisms of blood pressure regulation. Molecular Systems Biology.

[52] Jiang P (2015). A Systems Approach Identifies Networks and Genes Linking Sleep and Stress: Implications for Neuropsychiatric Disorders. Cell Reports.

[53] Schwartz SM, Schwartz HT, Horvath S, Schadt E, Lee S-I (2012). A Systematic Approach to Multifactorial Cardiovascular Disease. Arteriosclerosis, Thrombosis, and Vascular Biology.

[54] Peters J, Janzing D, Scholkopf B (2011). Causal Inference on Discrete Data Using Additive Noise Models. IEEE Transactions on Pattern Analysis and Machine Intelligence.

[55] Peters, J., Janzing, D. & Schölkopf B. *Elements of causal inference: foundations and learning algorithms*. (The MIT Press., 2017).

[56] Pearl, J. *Causality*. (Cambridge University Press, 2009).

[57] Cussens J (2014). Integer Programming for Bayesian Network Structure Learning. Quality Technology & Quantitative Management.

[58] Devasia, J. V. & Chandran, P. Inferring disease causing genes and their pathways: A mathematical perspective. arXiv:1611.02538. (2016).

[59] Quek, L.-E. & Nielsen, L. K. A depth-first search algorithm to compute elementary flux modes by linear programming. BMC Systems Biology **8** (2014). 10.1186/s12918-014-0094-2 PMC4236763 25074068

[60] Jindalertudomdee J, Hayashida M, Akutsu T (2016). Enumeration Method for Structural Isomers Containing User-Defined Structures Based on Breadth-First Search Approach. Journal of Computational Biology.

[61] Tang X, Wang J, Li M, He Y, Pan Y (2014). A Novel Algorithm for Detecting Protein Complexes with the Breadth First Search. BioMed Research International.

[62] Janzing D, Steudel B (2010). Justifying Additive Noise Model-Based Causal Discovery via Algorithmic Information Theory. Open Systems & Information Dynamics.

[63] Nonlinear causal discovery with additive noise models. *Advances in Neural Information Processing Systems*. (The MIT Press., 2017).

[64] Xiong, M. Big data in omics and imaging: integrated analysis and causal inference. (CRC Press, 2018).

[65] Parascandolo, G., Kilbertus, N., Rojas-Carulla, M. & Schölkopf, B. Learning Independent Causal Mechanisms. In Proceedings of the 35th International Conference on Machine Learning (ICML), **80**, pages: 4033–4041, Proceedings of Machine Learning Research, (Editors: Dy, Jennifer and Krause, Andreas), PMLR (2018).

[66] Wang, P., Rahman, M., Jin, L. & Xiong, M. A new statistical framework for genetic pleiotropic analysis of high dimensional phenotype data. *BMC Genomics* **17** (2016). 10.1186/s12864-016-3169-1 PMC5100198 27821073

[67] Boyd S (2010). Distributed Optimization and Statistical Learning via the Alternating Direction Method of Multipliers. Foundations and Trends® in Machine Learning.

[68] Parikh N (2014). Proximal Algorithms. Foundations and Trends® in Optimization.

[69] Udell M, Horn C, Zadeh R, Boyd S (2016). Generalized Low Rank Models. Foundations and Trends® in Machine Learning.

[70] Kalisch, M., Mächler, M., Colombo, D., Maathuis, M. H. & Bühlmann, P. Causal Inference Using Graphical Models with the R Packagepcalg. *Journal of Statistical Software* **47** (2012).

[71] Hauser, A & Bühlmann, P. Characterization and greedy learning of interventional Markov equivalence classes of directed acyclic graphs. arXiv:1104.2808 (2012).

[72] Yokoyama, A. S., Rutledge, J. C. & Medici, V. DNA methylation alterations in Alzheimer’s disease. *Environmental**Epigenetics* **3** (2017). 10.1093/eep/dvx008 PMC5804548 29492310

