# Supplementary material for: Shared Causal Paths underlying Alzheimer’s dementia and Type 2 Diabetes
Source: Sci Rep. 2020 Mar 5;10:4107. doi: 10.1038/s41598-020-60682-3 (PMC7058072; doi:10.1038/s41598-020-60682-3)
Supplement: Supplementary file 1 — Supplementary information. [file 41598_2020_60682_MOESM1_ESM.pdf]

Supplementary information for: "Shared Causal Paths underlying Alzheimer's dementia and Type 2 Diabetes"

Authors: Zixin Hu, Rong Jiao, Panpan Wang, Yun Zhu, Jinying Zhao, Phil De Jager ,David A Bennett, Li Jin and Momiao Xiong

Table S1. P-values of causation and association of 8 both directly and indirectly connected to both TD and T2DM.

	T2DM		AD	
**Gene**	**Causation**	**Association**	**Causation**	**Association**
GTF2H2C	<E-06	0.000156	<E-06	0.075045
NAIP	<E-06	0.000785	<E-06	0.057792
RN7SL9P	<E-06	0.000156	<E-06	0.075045
RP11-497H16.5	<E-06	0.001671	<E-06	0.182268
RP11-497H16.6	<E-06	0.000334	<E-06	0.200074
SERF1B	<E-06	0.000447	<E-06	0.163819
SMN2	<E-06	0.000447	<E-06	0.163819
ZNF658B	<E-06	0.867831	<E-06	0.102522

Table S2. P-value of 13 indirectly connected to AD and both directly and indirectly connected to T2DM causing genes.

	AD		T2DM	
**gene**	**Causation**	**Association**	**Causation**	**Association**
LINC01123	<E-06	0.2090558	<E-06	0.00782173
ZBTB45P1	<E-06	0.2090558	<E-06	0.00782173
GTF2H2C	<E-06	0.04533735	<E-06	0.00015593
NAIP	<E-06	0.02356008	<E-06	0.00078518
RN7SL9P	<E-06	0.07504514	<E-06	0.00015593
RP11-497H16.5	<E-06	0.02724587	<E-06	0.00167078
RP11-497H16.6	<E-06	0.10619584	<E-06	0.00033402
SERF1B	<E-06	0.01775603	<E-06	0.0004467
SMN2	<E-06	0.01775603	<E-06	0.0004467
RN7SL763P	<E-06	0.2426085	<E-06	0.88758712
RANP9	<E-06	0.15309853	<E-06	0.20641879
ZNF658B	<E-06	0.00674495	<E-06	0.20367155
AC132872.2	<E-06	0.11890796	<E-06	0.04748469

Table S3. P-value of 20 both directly and indirectly connected to AD and indirectly connected to T2DM causing genes.

	AD		T2DM	
**gene**	**Causation**	**Association**	**Causation**	**Association**
IGKV6D-21	<E-06	0.00936		0.00838
RN7SL632P	<E-06	0.00053		0.10824
CDH18	<E-06	0.00736		0.11987
HLA-DRB5	<E-06	0.01958		0.01317
RPL3P2	<E-06	0.00391		0.05737
FAM74A6	<E-06	0.00256		0.17522
RNU6-156P	<E-06	0.00209		0.00086
RP11-15J10.1	<E-06	0.00876		0.01246
RP11-262H14.4	<E-06	0.00173		0.01412
RP11-318K12.2	<E-06	0.0031		0.00086
RNU6-702P	<E-06	0.01116		0.26546
AL021920.1	<E-06	0.00529		0.10803
AL590452.1	<E-06	0.0034		0.01512
EIF1AXP1	<E-06	0.00529		0.10803
FAM231B	<E-06	0.00187		0.28261
PRAMEF11	<E-06	0.00122		0.07388
RP5-845O24.8	<E-06	0.00122		0.07388
TTC3	<E-06	0.0102		0.01343
AC009237.16	<E-06	0.0024		0.17164
AC009237.17	<E-06	0.0024		0.17164

Table S4. A total of 19 gene expressions caused AD.

	AD		T2DM	
**gene**	**Causation**	**Association**	**Causation**	**Association**
**PLA2G15**	<0.0001	0.0012	0.6633	0.7645
**GRPEL2**	<0.0001	0.0026	0.3611	0.9894
**TCEAL6**	<0.0001	0.0001	0.7324	0.3729
**C19orf71**	<0.0001	0.0002	0.8063	0.4285
**GRTP1**	<0.0001	0.0004	0.7326	0.4044
**CTC-471C19.1**	<0.0001	<0.0001	0.9039	0.8677
**GAP43**	<0.0001	0.0001	0.413	0.2183
**NTNG1**	<0.0001	<0.0001	0.2216	0.298
**BIN1**	<0.0001	0.4087	0.9583	0.809
**ZNF683**	<0.0001	0.0098	0.5699	0.391
**PIGS**	<0.0001	0.0624	0.361	0.7423
**MRPS18A**	<0.0001	0.0004	0.4478	0.8081
**PRKCD**	0.0001	0.0006	0.5847	0.0814
**FNDC5**	0.0001	0	0.8283	0.9253
**SLC7A14**	0.0001	0.0101	0.9696	0.7183
**GRAMD1B**	0.0001	0.0171	0.0005	0.6413
**USP12**	0.0001	0.041	0.5937	0.0269
**DHRS7**	0.0001	0.0571	0.9111	0.4734
**OAZ1**	0.0001	0.0089	0.7556	0.6632

Table S5. A total of 7 gene expressions causing T2DM, but did not cause AD.

	**AD**		**T2DM**	
**gene**	**Causation**	**Association**	**Causation**	**Association**
**GRN**	0.3019	0.3037	<0.0001	<0.0001
**ATP1B3**	0.3848	0.3358	<0.0001	<0.0001
**CTD-3065J16.6**	0.5351	0.2133	<0.0001	0.4403
**BX571672.1**	0.587	0.3088	<0.0001	0.5182
**SLC25A35**	0.7881	0.3619	<0.0001	0.6975
**PRR12**	0.9786	0.281	<0.0001	0.3704
**AOC3**	0.9936	0.0678	<0.0001	0.1873

Table S6. A list of 17 methylated sites/genes that caused AD.

Methylated Gene	Causation	Association
EMX2OS	< 1e-4	0.1973
PIPOX	< 1e-4	0.6245
DHX8	< 1e-4	0.7317
cg04413644	< 1e-4	0.0086
cg12049093	< 1e-4	0.1122
cg21346589	< 1e-4	0.235
cg18527583	< 1e-4	0.0126
cg00639635	< 1e-4	0.2032
cg22133973	< 1e-4	0.0004
cg12644659	< 1e-4	0.7813
cg12949927	< 1e-4	0.0915
cg20714487	< 1e-4	0
cg03701930	< 1e-4	0.5572
CARKD	< 1e-4	0.2309
NYNRIN	< 1e-4	0.1213
IL2RA	< 1e-4	0.8899
cg02438164	< 1e-4	0.0029

Table S7. A list of 27 methylated sites/genes that caused T2DM, but did not cause AD.

Methylated Gene	Causation	Association
cg27424148	< 1e-4	0.8752
MIR220B	< 1e-4	0.3568
cg16991316	< 1e-4	0.0292
cg01433468	< 1e-4	0.4165
cg04124260	< 1e-4	0.0587
cg06769739	< 1e-4	0.0004
cg11989330	< 1e-4	0.0006
cg17883371	< 1e-4	0.0075
GSTTP1	< 1e-4	0.0016
YWHAQ	< 1e-4	0.6393
cg25004193	< 1e-4	0.0001
cg25757820	< 1e-4	0.2169
cg02093808	< 1e-4	0.2378
C11orf45	< 1e-4	0.1566
cg00850073	< 1e-4	0.0136
NME5	< 1e-4	0.1576
cg14727987	< 1e-4	0.0011
cg10245123	< 1e-4	0.0014
MFHAS1	< 1e-4	0.5626
cg11388673	< 1e-4	0.6857
cg18001780	< 1e-4	0.0127
KRT77	< 1e-4	0.0291
cg16624888	< 1e-4	0
TPT1	< 1e-4	0.2871
CCDC70	< 1e-4	0.0186
cg01358406	< 1e-4	0.0119
cg03608502	< 1e-4	0.1001

Table S8. A list of 120 DNA methylation sites/genes that were indirectly connected to AD and T2DM.

Methylation Site/Gene	Expressed Genes	Causation	Association
cg18747197	QKI	0.00004	
cg19351026	GABRA2	0.00018	0.0000101
cg27665808	snoU13	0.00014	0.0000117
GSTTP1	RP11-259G18.2	0.00007	0.0000155
cg12363375	CTD-3025N20.2	0.00021	0.0000198
cg16677162	GPX6	0.00011	0.0000224
cg13401079	RCOR1	0.00015	0.0000277
cg17670237	RP11-259G18.2	0.00015	0.0000277
cg07536144	U1	0.00001	0.0000458
ABCA10	RP11-86H7.7	0.0001	0.0000464
cg22463795	GABRA2	0.00019	0.0000465
cg01797450	ASS1	0.00008	0.0000503
cg23454003	PSMB6	0.00009	0.0000545
cg05455747	ZC3H4	0	0.0000623
cg17526301	GPX6	0.00003	0.0000806
cg10061805	PPIAP2	0.00018	0.000152878
cg02190400	ZC3H4	0.00005	0.000166344
cg15304404	RXFP4	0.00028	0.000182858
cg24751928	CTD-2501E16.2	0.00004	0.00019633
cg17125990	GABRA2	0.00003	0.000205598
cg00415011	GARNL3	0	0.000208346
cg23340218	WNT3A	0.00003	0.000244508
cg26274929	UTP6	0.00005	0.00026591
cg09977969	CTD-2116F7.1	0.0002	0.000268455
cg02461269	CTD-3025N20.2	0.00006	0.000315482
cg02523270	GARNL3	0.00036	0.00032395
cg09550810	GARNL3	0.00002	0.000380359
cg07475973	RP11-265D19.6	0.00012	0.000391326
cg21686890	ABCB6	0.00002	0.000398401
cg21099148	snoU13	0.00019	0.000425933
cg01870681	GPX6	0.00019	0.000440409
cg25112877	WNT3A	0.00014	0.000463058
cg19711815	RP11-259G18.2	0.00008	0.000548779
cg06447341	TTC37	0.00003	0.000685772
ch.2.3048096R	ASS1	0.00002	0.000716838
cg07536144	PLCH1-AS1	0.00009	0.000744448

cg15426660	snoU13	0.00001	0.000760849
cg08209099	RP11-259G18.2	0.00007	0.000785023
cg22891595	GARNL3	0.00001	0.000870647
cg06181286	CBLN4	0	0.000984836
cg14739859	RP11-259G18.2	0.00005	0.000989198
cg12548341	GNAL	0.00013	0.001060884
cg15426660	SNTB2	0.00009	0.001162623
cg10059756	GABRA2	0.00004	0.001207758
cg21205865	snoU13	0.00004	0.00131807
cg00039801	RP11-259G18.2	0.00006	0.001347476
cg10316834	snoU13	0.0001	0.001351471
cg20078879	RPS4X	0.00009	0.001369325
cg24015081	CTD-2176I21.1	0.00018	0.00144952
cg25214310	SUGT1	0.00037	0.001534327
cg14687029	ABCB6	0.00008	0.001560267
cg05157340	RANBP2	0.00014	0.00199909
cg06709828	CTD-3025N20.2	0	0.00207517
cg06112654	SNTB2	0.00004	0.002087027
cg06112654	snoU13	0.00006	0.002181983
cg22651787	FAM188B	0.00005	0.002497132
cg14577406	RBPMS2	0	0.002576388
cg04854637	SUGT1	0.00003	0.003268184
cg12180270	GABRA2	0.00002	0.003306246
cg04606076	RXFP4	0.00012	0.00331699
cg02486332	CASP12	0.00012	0.003429848
cg11868247	RP11-351K16.4	0.00007	0.003504288
cg13001963	RXFP4	0	0.00399057
cg24430419	snoU13	0.0001	0.004028793
cg07716131	SAA1	0.00016	0.004568173
MIR145	RCOR1	0.00003	0.004796959
cg16101962	GARNL3	0.00002	0.00494505
cg10238080	snoU13	0.00063	0.005006355
cg12094552	SNTB2	0.00005	0.005246665
cg23346544	ARHGAP20	0	0.005555473
cg13332807	PKN2	0.00013	0.006109406
TDGF1	ST6GALNAC6	0.00005	0.007245283
cg24617444	RUSC1	0	0.007756879
ANKRD53	CPE	0.00021	0.007916803
cg20918393	PPIAP2	0.00009	0.008203878
cg23925513	RARB	0.00012	0.008650139
cg18105749	BRSK2	0.00005	0.009148187

cg16624888	RXFP4	0.00006	0.009483327
cg09315586	SUGT1	0.00001	0.009806715
cg20078879	DNAJC1	0.00007	0.010129593
cg00651087	ZNF114	0.00005	0.011173318
cg04606076	C2CD2L	0.00015	0.012211784
cg10061805	FAN1	0.00019	0.012503808
cg25900943	SNTB2	0.00001	0.013080748
HRCT1	DCTN2	0.00028	0.01314581
cg05521150	PMS2CL	0.00005	0.014411814
cg11868247	CSNK1G1	0.00007	0.014603357
cg11868247	EIF6	0.00006	0.016552782
cg24631482	LA16c-444G7.2	0.00005	0.017664588
cg04858776	OR2L13	0.00015	0.018602077
cg15022039	SRD5A1	0.00033	0.018987855
C6orf223	SMO	0.00003	0.020149091
cg17624891	DGKK	0.00007	0.020430983
cg25816127	WNT3A	0	0.020805286
cg26686150	FAM213B	0.00001	0.021353401
cg19937979	hsa-mir-146a	0.00002	0.021459718
cg04819760	FAH	0.00057	0.022816943
cg07929768	POTEG	0.00016	0.023349672
cg26095395	ADRA1B	0	0.025356864
cg14145524	XRCC5	0	0.025570462
cg17214023	INPP5F	0.00008	0.025878158
cg16609139	SDS	0.00013	0.027422099
ch.11.96774805R	RPS4X	0.00003	0.027946801
ch.10.109266902R	RP11-351K16.4	0.00013	0.029508409
cg02796279	OR7C1	0.00004	0.032915515
cg19937979	NDUFA8	0.00003	0.034228407
ch.2.1894803R	NUMB	0.00006	0.034430887
cg14257429	SNTB2	0.00002	0.036581547
cg08122831	RP11-284N8.3	0.00001	0.037802122
cg11465213	EXOSC2	0.00006	0.040519116
cg05766107	ADCYAP1R1	0.00005	0.040760293
cg00895132	ZNF233	0.00003	0.041852386
cg19355069	ESD	0.00014	0.043231379
cg18978531	C2orf18	0.00009	0.047170478
MYCT1	RP11-265D19.6	0.00002	0.047607389
cg03257930	FADS6	0.00003	0.048071635
cg11540476	RP11-114F3.5	0.00001	0.048588166

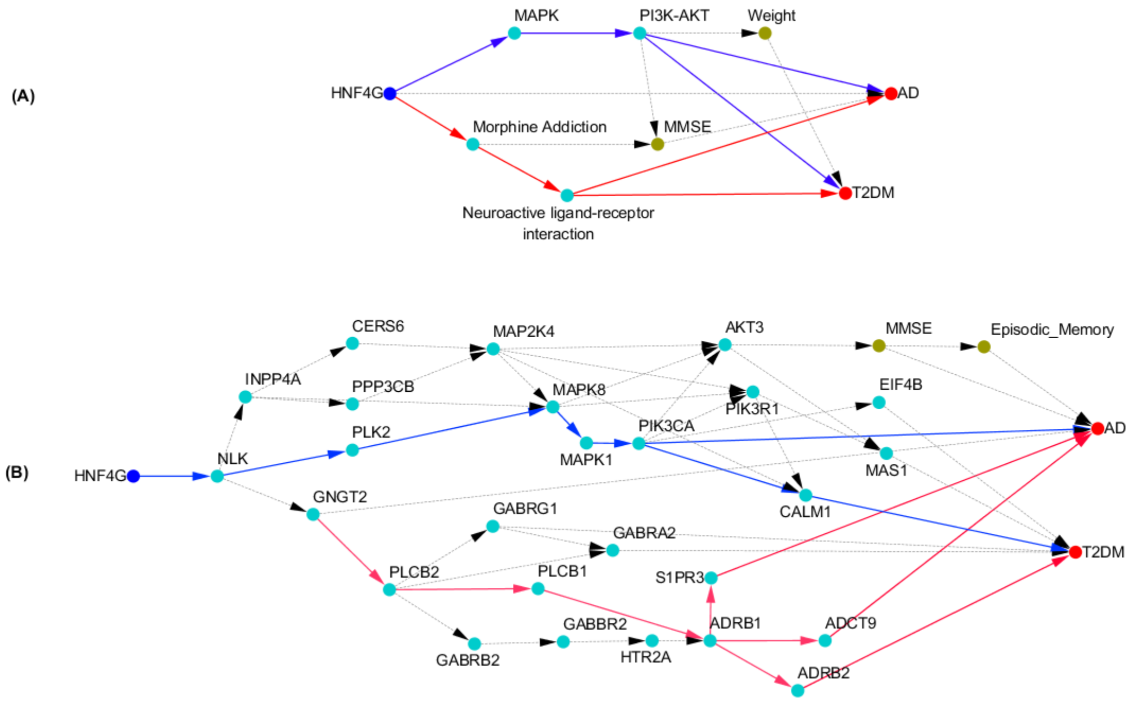

Figure S1. (A) Shared morphine addiction and neuroactive ligand receptor interaction pathways between AD and T2DM; (B) Shared causal subnetwork structure from HNF4G to AD and T2DM.

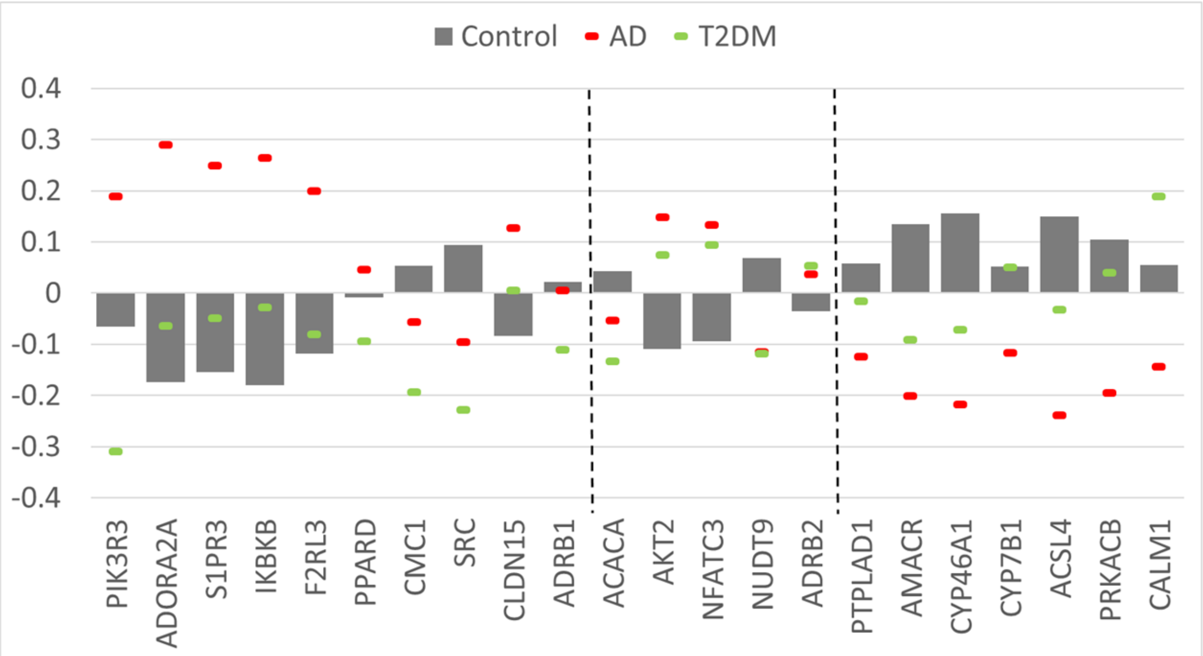

Figure S2. Average expression levels of genes in Figure 3 for AD, T2DM and normal individuals where gene expression levels were normalized.

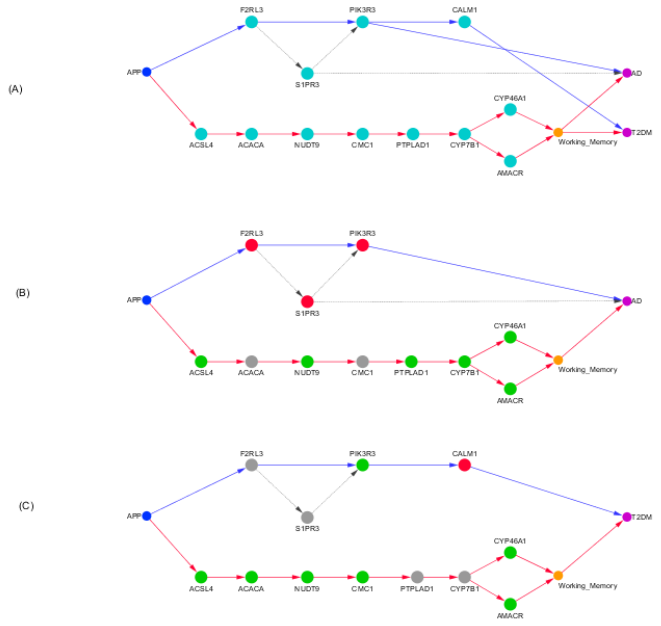

Figure S3. (A) Two major causal paths from APP to AD and T2DM in Figure 3; (B) Differential expressions of the genes along two major causal paths in (A) between AD and normal individuals where the nodes in red color represented over expressed genes, the nodes in green color represented the under expressed genes and the nodes in the grey color represented the genes showing no differential expressions; (C) Differential expressions of the genes along two major causal paths in (A) between T2DM and normal individuals where the nodes in red color represented over expressed genes, the nodes in green color represented the under expressed genes and the nodes in the grey color represented the genes showing no differential expressions.
